# High performance p-channel and ambipolar OFETs based on imidazo[4,5-*f*]-1,10-phenanthroline-triarylamines†

**DOI:** 10.1039/d0ra00210k

**Published:** 2020-03-31

**Authors:** Ramachandran Dheepika, Ramakrishnan Abhijnakrishna, Predhanekar Mohamed Imran, Samuthira Nagarajan

**Affiliations:** Department of Chemistry, Central University of Tamil Nadu Thiruvarur-610 005 India snagarajan@cutn.ac.in; Department of Chemistry, Islamiah College Vaniyambadi-635 752 India

## Abstract

A series of phenanthroline functionalized triarylamines (TAA) has been designed and synthesised to evaluate their OFET characteristics. Solution processed OFET devices have exhibited p-channel/ambipolar behaviour with respect to the substituents, in particular methoxyphenyl substitution resulted with highest mobility (*μ*_h_) up to 1.1 cm^2^ V^−1^ s^−1^ with good *I*_on/off_ (10^6^) ratio. These compounds can be potentially utilized for the fabrication of electronic devices.

## Abstract 2

A series of phenanthroline functionalized triarylamines (TAA) has been designed and synthesised to evaluate their OFET characteristics. Solution processed OFET devices have exhibited p-channel/ambipolar behaviour with respect to the substituents.

## Graphical abstract

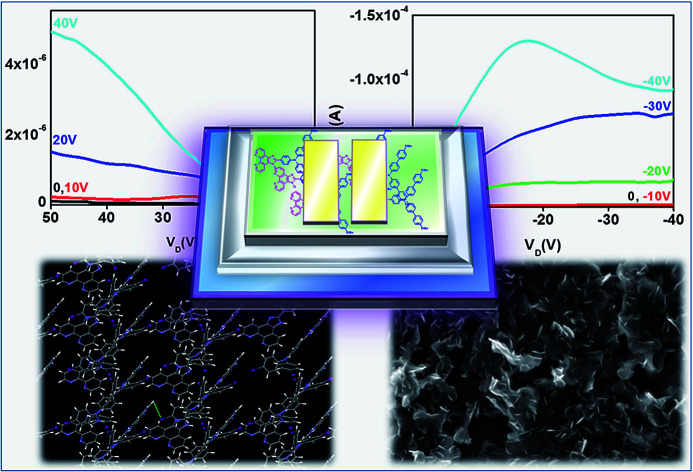

## Introduction

Organic field effect transistors (OFETs) unquestionably are the candidates of future electronics owing to their flexibility which is very essential for a variety of applications including artificial human skin.^1^ In the past decade OFETs have been exploited for radio frequency identification tags, sensors, integrated circuits, logic switches, and larger flexible display applications.^2^ Solution processing based small molecule are studied due to their facile and economic methodologies which enables the commercialization of organic electronic devices. Among the molecules studied, pentacene, rubrene and polythiophene have exhibited astonishingly good transistor characteristics.^3^ However, the aforementioned molecules are classic p-channel materials; n-channel molecules face problems in terms of stability because of their low lying LUMO levels and are lagging behind in the race.^4^ Ambipolar devices will integrate electronic properties of both p and n channel molecules in a single device.^5^ From a performance perspective complementary logic is also crucial to achieve high speed with low power dissipation to ensure low noise margin and robust operation of devices such as inverters and memory elements.

The ambipolar devices can be obtained from a fused D–A system or D and A co-systems. Donor–acceptor hybrids have gained importance owing to their intramolecular electron transfer nature which can be tuned/altered by external stimulus.^6,7^ D–A systems, in terms of OFET have grabbed enormous attention in order to make ambipolar OFETs^8^ for integrated circuits and inverters. Although, the unipolar devices are more appealing, the covalent interaction between the donor and acceptor leads to optimized band gap which allows them to unveil their balanced p and n channel behaviour.

A few combinations of TAA and phenanthroline have been analysed for their application in OLEDs,^9^ linear and non-linear optics and guest–host systems.^10^ In addition, phenanthrolines were investigated by coordination chemists due to its perfectly placed N atoms. Imidazole fragment, in organic frame work is acknowledged to exert substantial inspiration in inter and intramolecular interactions.^11^ Although, TAAs are investigated for their application in OFETs and resulted in good mobility and ON/OFF ratio,^12,13^ to the best of our knowledge, OFETs based on phenanthrolines have not been reported yet.

Herein, we have designed and synthesised a series of new fused D–A systems for OFET application with TAA served as donor and phenanthrolines as acceptor. The OFET characteristics with respect to the substituents are scrutinised. In one arm of TAA electron accepting, planar phenanthroline is attached through an azole ring. This substitution tunes the bandgap by altering the frontier molecular orbitals and can facilitate charge carrier movement. Solution processing from a chloroform solution assisted the molecule to self-assemble efficiently to improve the communication among the molecules. Post thermal annealing gave highly crystalline thin film. This investigation can give an insight about tuning the OFET behaviour of TAAs and the effect of substituents.

## Experimental

### Materials and methods

1,10-Phenanthroline, triphenylamine, CH_3_COONH_4_, NaBr, and the solvents used for synthesis are purchased from commercial resources and used as received. ACS grade solvents were used for analysis. Nuclear magnetic spectroscopic (NMR) characterisation of the compounds were done in a Bruker 400 MHz spectrometer. TMS was used as standard in d^6^-DMSO/CDCl_3_. The molecules were further analysed by Thermo Exactive Plus mass spectrometric technique. UV-visible absorption spectra were recorded in JASCO UV-NIR spectrophotometer. Emission behaviour was investigated using PerkinElmer Spectrofluorimeter LS 55. Electrochemical work station (CHI 6035D) was utilised to study the electrochemistry of the molecules. PerkinElmer TGA 4000 instrument was utilised to study the thermal behaviour of the compounds by thermogravimetric analysis.

Mass spectra was recorded in positive scan mode in methanol as solvent. Photophysical studies were carried out in dichloromethane at room temperature. The concentration was fixed at 10^−5^ M for absorption and 10^−7^ M for emission studies. For electrochemical characterizations, classic three electrode set up was used with glassy carbon working electrode and standard calomel electrode as reference electrode. To complete the circuit platinum wire was used as the counter electrode. The electrode was polished before each experiment with alumina slurry and ferrocene was used as standard. Tetrabutylammonium hexafluorophosphate was used as supporting electrolyte. The solution was purged with N_2_ before each experiment to remove oxygen inference. Thermal analysis were done in nitrogen atmosphere at the heating rate of 10 °C per minute. DFT and TD-DFT methods have been used to understand the theoretical aspects.

### Synthetic procedure

Sulphuric acid (20 mL) was stirred with 1,10-phenanthroline (1.01 mmol) in an ice bath for 10 min. To the stirring mixture NaBr (11.08 mmol) was added in several small portions. The reaction mixture was stirred for 5 min in ice and followed by slow addition of HNO_3_ (10 mL) and continued to stir for 20 min in ice bath. Temperature was increased to reach 100 °C. After 3 hours, the reaction was quenched and poured into crushed ice. Neutralization with NaHCO_3_ to pH: 6–7 yielded the product and extracted using CH_2_Cl_2_. The organic layer was dried over sodium sulphate and concentrated to give a yellow orange solid of 89% yield after recrystallization from ethanol.

Triarylamine aldehydes were synthesised according to the procedure reported in our previous work.^13^ A mixture of 1,10-phenanthroline-5,6-dione (5 mmol), ammonium acetate (100 mmol) and corresponding TAA aldehydes (6 mmol) was dissolved in glacial acetic acid (4 mL), heated at 100 °C for 20 min in CEM microwave synthesizer (power: 150 W, pressure: 150 psi, pre-stirring: 2 min) and monitored. After completion the reaction mixture was poured into ice to get a yellow precipitate and collected by filtration (Whatmann 40). The precipitate was neutralised with concentrated ammonia solution and followed by hot water wash. The precipitate was dried to get a yellow solid in 95–98% yield. The crude product was recrystallized from ethanol and DMF mixture.

### Device fabrication

Bottom gate top contact architecture has been chosen for robust device fabrication by solution processing. n^++^ doped silicon wafer was used as substrate with thermally grown silicon dioxide layer as dielectric layer. Si wafer was pre-cleaned with standard cleaning procedures before the fabrication. The Si wafer acts as gate electrode. The device architecture is shown Fig. S1.† The compounds were dissolved in chloroform (5 mg mL^−1^) and sonicated for 20 minutes. The prepared sample solution was spun over the dielectric layer in 1600 rpm for 60 s. Then the device was soft baked and followed by annealing at 80 °C for 40 minutes. To complete the fabrication source and drain contacts were placed through a mask with channel length of 150 μm and width of 5 mm. Transistor characteristics were acquired using a Keithley 4200 SCS semiconductor parameter analyzer system.

## Results and discussion

**Scheme 1:**
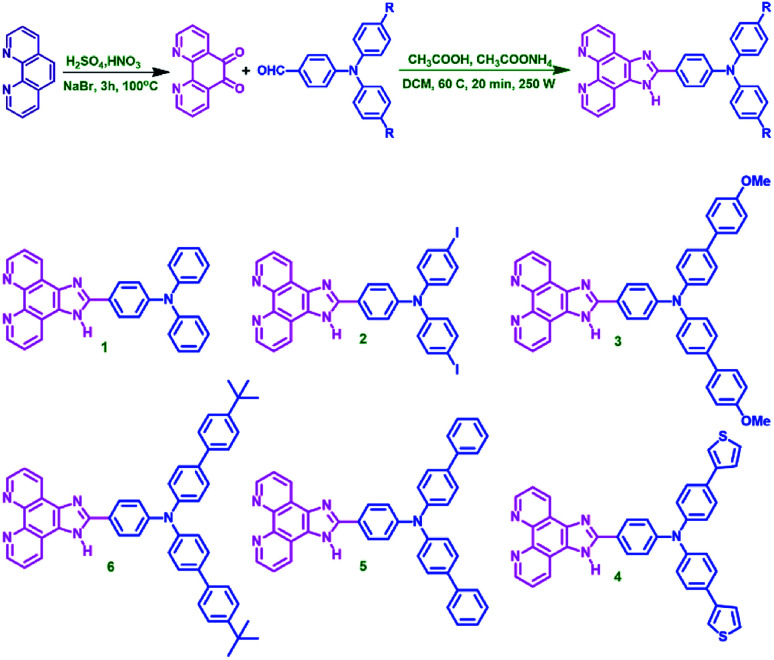
Synthetic route and the structures of compounds 1–6.

The target molecules are synthesised *via* microwave assisted green synthesis with high purity and yield. The synthetic sequence and the molecular structure are shown in Scheme 1. The compounds are characterised well by various techniques. In ^1^H NMR, the N–H proton is observed as high de-shielded proton at around 13 ppm and the aromatic protons are resonating in 7 to 10 ppm region. In compound 3, methoxy proton is observed at 3.865 ppm and in compound 6*t*-butyl group protons are resonating at 1.321 ppm. ESI mass analysis in positive scan mode has resulted in [M + H]^+^molecular ion peak as base peak for all the molecules. Thermal analysis, device fabrication, thin film characterizations and computational analysis are discussed in detail in ESI.†

### Photophysical studies

**Fig. 1:**
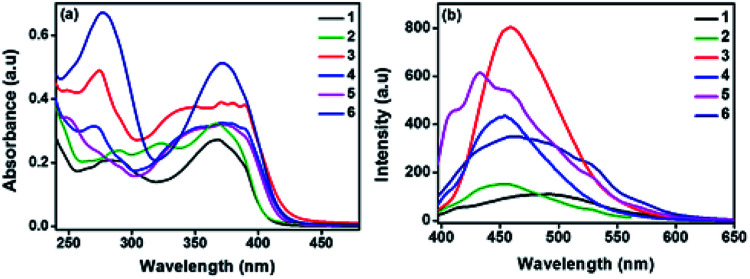
(a) Absorption and (b) emission spectra of compounds 1–6.

To investigate the photophysical behaviour of the new molecules, UV-vis absorption and emission spectra were analysed. Spectra were recorded in dichloromethane (DCM) at 10^−5^ M concentration, given in the Fig. 1(a) and the data are represented in Table 1. The two peaks are attributed to n–pi* and pi–pi* transitions. Compounds 1 and 2 with relatively less conjugation than the other compounds, have lower absorbance (*ε* = 2.7 and 2.9 × 10^4^ M^−1^ cm^−1^). Compounds 3 and 4 have exhibited red shift in their lower energy absorption. This may be due to the electron rich nature of the OMe-phenyl and thiophene substituents attached to TAA arms. Molecule 6 exhibited the highest absorbance (*ε* = 5.1 × 10^4^ M^−1^ cm^−1^). In addition, TD-DFT calculations informs that the S_0_–S_1_ transition is most feasible for all the compounds with regard to the oscillator strength (Table S6†). The emission spectra were recorded in DCM at 10^−7^ M concentration and the spectra are represented in Fig. 1(b). All the molecules exhibited broad peaks covering from 400–600 nm. The shape of the emission spectra are not mirror images of the absorption spectra, which implies the geometrical changes in the excited state of the molecule. In polar medium, excited state of the molecules gain more dipole moment due to the charge separation within the molecule.^14^ In addition, all the molecules have significant Stokes shift values and are given in Table 1. Compound 3 exhibited the higher intensity, may be due to the chromophoric effect of OCH_3_ group. From DFT studies, with respect to the substituents the planarity of the molecule changes from ground state to excited state. This observation is consistent with regard to the dihedral angle of two nitrogen centres (TAA and azole ring) (Fig. S2 and Table S7†).

**Table 1:** Photophysical properties of compounds 1–6

Comp. no.	*λ*_abs_ (nm)	*λ*_em_ (nm)	Stokes' shift (cm^−1^)	Absorption co-efficient *ε* (10^4^ M^−1^ cm^−1^)
1	282, 368	489	6724	2.7
2	288, 366	452	5199	2.9
3	274, 369	459	5171	3.9
4	270, 370	454	5001	3.2
5	370	433	3933	3.2
6	277, 371	463	5356	5.1

### Electrochemistry

**Fig. 2:**
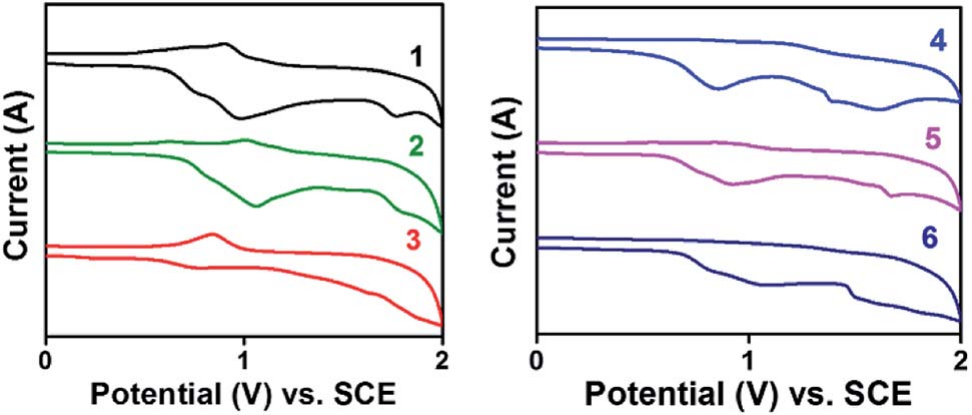
Cyclic voltammogram of compounds 1–6.

Electrochemical behaviour of the new molecules were investigated by cyclic voltammetry technique in a classic three electrode cell set up. Tetrabutylammonium hexafluoro phosphate was used as supporting electrolyte in *N*,*N*′ dimethyl formamide (DMF) at the scan rate of 50 mV s^−1^. Irreversible redox peak was observed for all the compounds in positive potential region. The solution was purged with nitrogen for 15 min to eliminate the interference of dissolved oxygen in the system. HOMO levels were calculated with reference to ferrocene using the formula *E*_HOMO_ = −(*E*_ox_ + 4.8 − *E*_fc/fc^+^_).^15^ The values fall in the range of −5.2 to −5.6 eV which is matching with widely used hole transporting and electron blocking layers.^16^ This HOMO values ensure that these molecules can be effectively employed in various electronic devices including solar cells, light emitting diodes and transistors. Band gap (optical *E*_g_) was calculated from absorption onset and corresponding LUMO values were obtained. Compounds 1 and 2 have band gap at 3.0 eV may be due to the insignificant role of –H and –I substituents in altering the FMOs position. Introducing electron rich thiophene ()4 and methoxyphenyl ()3 groups in TAA moiety have tuned the band gap to 2.95 and 2.96 eV respectively. The irreversible redox peak observed in the range of 0.8 to 1.2 V corresponds to the TAA moiety (Table 2 and Fig. 2).

**Table 2:** HOMO, LUMO and band gap of compounds 1–6

C. no.	Experimental (eV)			Computational^a^ (eV)		
	HOMO	LUMO	Band gap	HOMO	LUMO	Band gap
1	−5.347	−2.307	3.040	−5.083	−1.433	3.649
2	−5.450	−2.387	3.063	−5.504	−1.893	3.610
3	−5.282	−2.328	2.954	−4.917	−1.435	3.482
4	−5.604	−2.604	2.960	−5.075	−1.527	3.548
5	−5.345	−2.356	2.989	−5.035	−1.489	3.546
6	−5.647	−2.676	2.971	−4.968	−1.452	3.515

a Obtained from DFT-(B3LYP 6-31(d)-G) method.

### Thermal behaviour

**Fig. 3:**
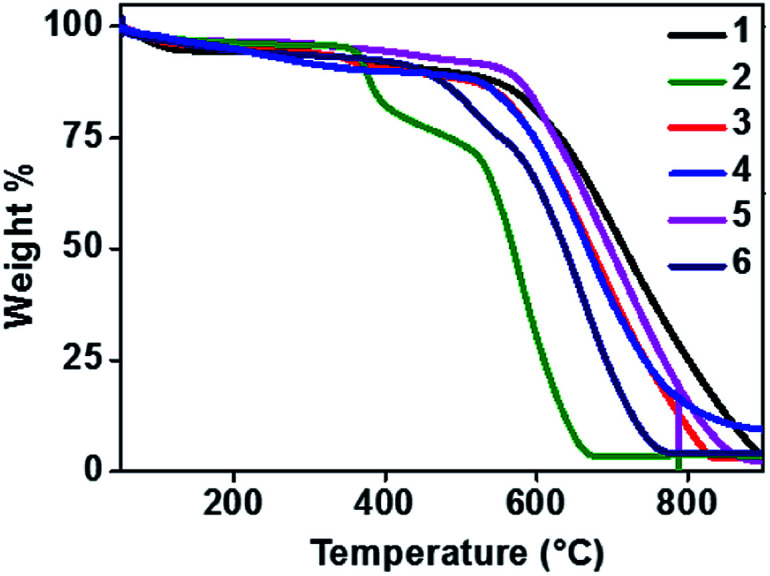
TGA curves of compounds 1–6.

Thermal stability of a semiconducting molecule is an imperative parameter to decide upon the lifetime and efficiency of the devices. All the new phenanthroline-TAAs were analysed by thermogravimetric (TGA) method to study their thermal characteristics. The experiments were carried out at 10 °C per minute heating rate in nitrogen atmosphere. The TGA curves are given in Fig. 3 and data are represented in Table 3.

**Table 3:** Thermal behaviour of compounds 1–6

Comp. no	*T*_m_ (°C)	*T*_d_ (°C)
1	343	475
2	330	375
3	374	427
4	295	392
5	376	560
6	356	450

Introducing a planar phenanthroline moiety in one arm of the TAA have noticeably improved the thermal stability and ensures efficiency. The melting point ranging from 295 to 376 °C and for compounds 3 and 5 the higher *T*_m_ (374 and 376 °C respectively) have been observed. Similarly, *T*_d_ corresponding to 10% weight loss is in the range of 375 to 560 °C. The decomposition temperature of these phenanthroline substituted TAAs are upgraded from previously reported TAAs.^19^

### Thin film characterization

**Fig. 4:**
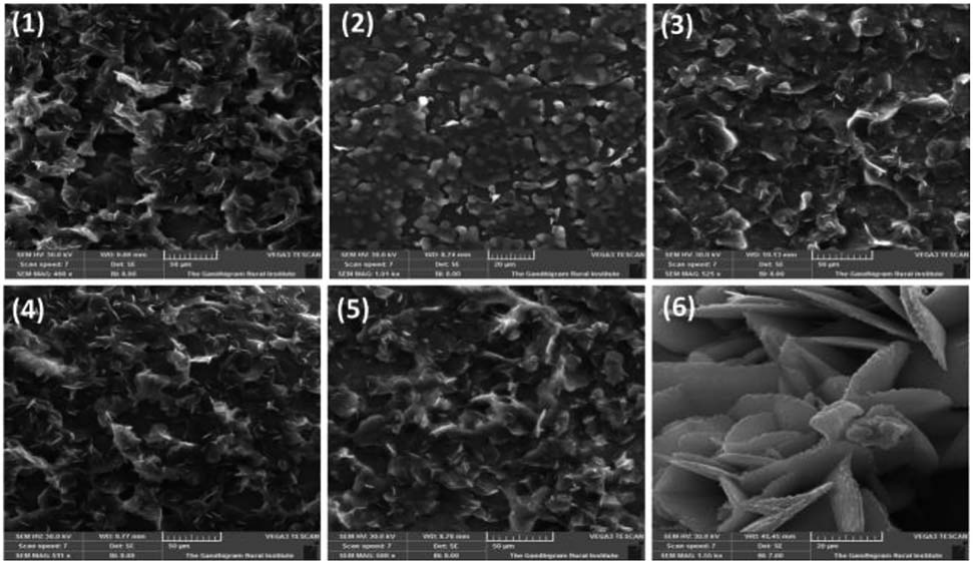
Thin film morphology SEM analysis.

Thin films of bottom gate top contact devices for compounds 1–6 are characterized by SEM and XRD analysis. Films are spin coated from chloroform solution and annealed at 80 °C for 40 minutes. Fig. 4 shows the morphology of the active thin film by SEM analysis. All the compounds have given good self-assembled crystalline thin film. However, in case of compound 2, blend granule like arrangements was observed but the assembly is not well connected may be due the presence of heavy atom. Thus this compound failed to produce any transistor characteristics. Compounds 1, and 3–5 shows uniform packing and almost similar arrangements with well-connected network. On comparison with reported TAA derivatives, self-assembly has changed significantly. In case of compound 3, the micro cluster is observed where the corresponding TAA aldehyde have exhibited spindle fibrous network.^13^ This observation describes the role of phenanthroline unit in altering the self-assembling nature of the TAAs. Interestingly, compound 6 shows petal like morphology may be due to the *t*-butyl group present in TAA side. *t*-Butyl group is known to play a vital role in supramolecular self-assembly of organic frameworks.^20^

**Fig. 5:**
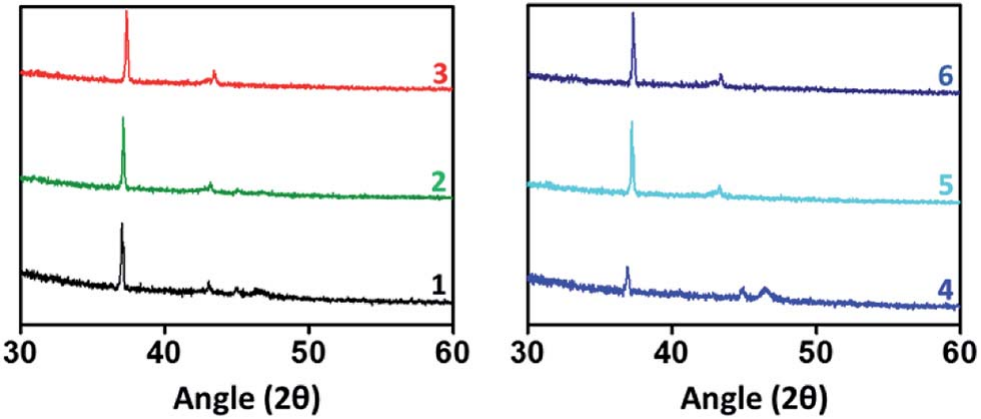
Thin film XRD analysis.

Thin film X-ray diffraction analysis have been carried out to study the film morphology. All the thin films were coated from a chloroform solution, spun in 1600 rpm for 60 seconds and thermally annealed at 80 °C to remove the residual solvents and to improve the self-assembly. The thin film XRD patterns are given in Fig. 5. All the compounds have produced highly crystalline film. In thin film, the compounds exhibited a diffraction peak around 2*θ* = 37° and 43° corresponding to d-spacing of 24 Å and 20 Å respectively.^22^ The peak at 37° was much sharper with a stronger intensity than the other peaks indicates that the orientation of the molecule was almost in the way of 37°.^23^ In addition, compounds 1 and 4 have exhibited an additional weak diffraction peak with d-spacing of 19.5 Å which indicates long range order.^24^ Crystallinity is one of the important criteria to attain good charge carrier mobility in thin film transistors.^21^ The average crystal size of the films were calculated to be in the range of 200–500 nm. The compound 4 has exhibited the low reflection at 2*θ* = 37°, may be due to the relatively small crystal size. Thermal annealing at 80 °C have changed the crystallinity and resulted in less intense diffraction peaks. The crystalline size must be uniform with less grain boundaries to avoid trapping of charges which deviates the charge carrier movement and ultimately reduces the performance of the device. Here, compounds have resulted in good crystalline film after annealing at 80 °C. It is important to note that without thermal annealing uniform film was not obtained. From SEM and XRD analysis, it is ensured that the constant microstructural construction of active thin film renders good charge carrier mobility in OFET device.

### OFET characteristics

**Fig. 6:**
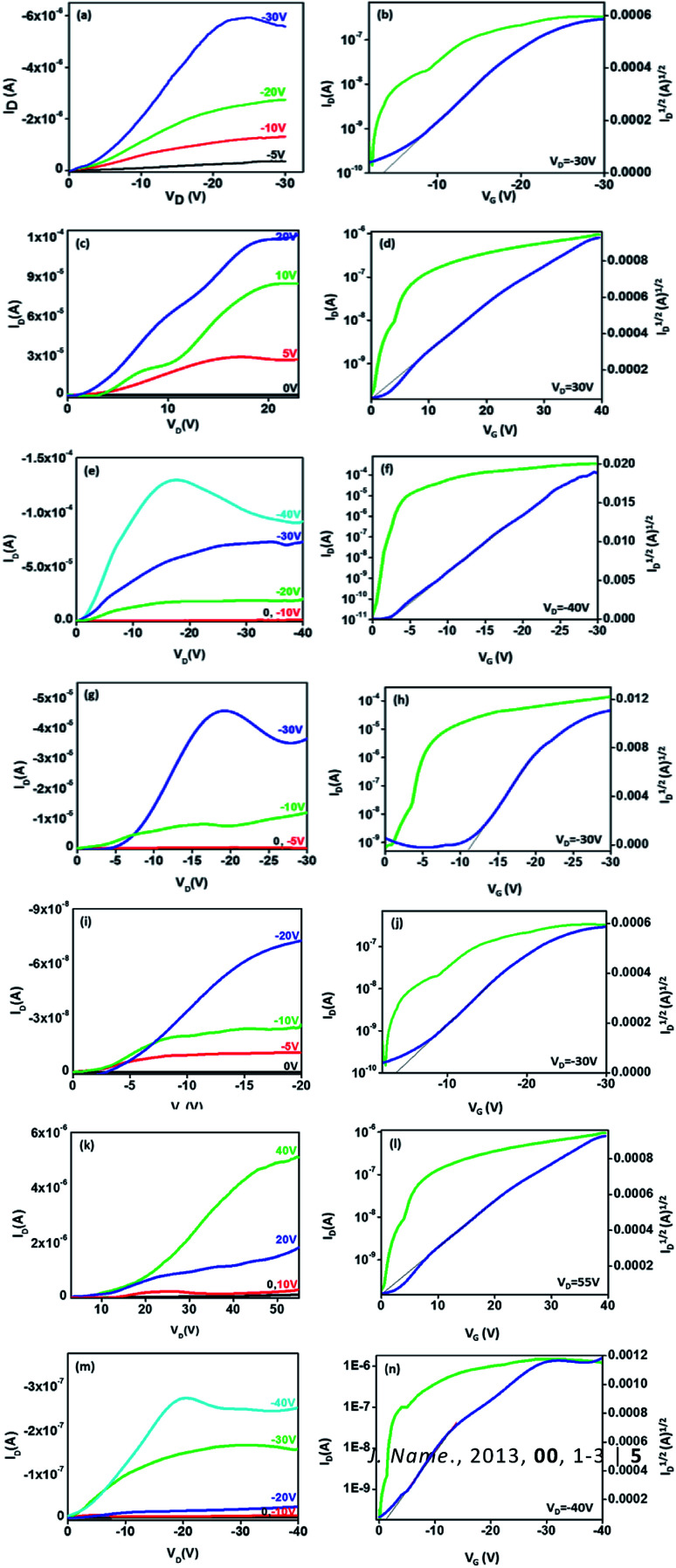
OFET characteristics of compounds 1 ((a–d), ambipolar) 3 ((e and f), p-channel), 4 ((g and h), p-channel) 5 ((i–l), ambipolar) and 6 ((m and n), p-channel) in transfer curves, log *I*_D_ and (*I*_D_)^1/2^ curves are represented in green and blue colour, respectively.

The bottom gate top contact devices are analysed for their transistor behaviour in ambient conditions. Among the compounds analysed, five compounds have exhibited transistor characteristics. Chloroform allows the molecule to self-assemble efficiently and the residual solvent can be removed easily by thermal annealing. Compounds 1 and 5 have shown bipolar charge carrier mobility whereas, compounds 3, 4 and 6 exhibited only p-channel behaviour (Fig. 6, Table 4). This observations can be accounted for the nature of substituents; electron rich methoxyphenyl, thiophene and *tert*-butylphenyl groups which improves the donating nature of the TAA moiety, thus p-channel behaviour is prominent and no n-channel behaviour is observed. The mobility is extracted from the saturation region using the formula,
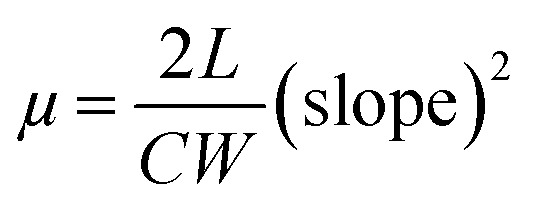
where, *C* is capacitance of the dielectric layer per unit area, *L* and *W* are length and width of the channel, respectively. Threshold voltage (*V*_TH_) is calculated from the plot of square root of *I*_DS_*vs.* gate voltage (*V*_G_). All the compounds exhibited mobility in 10^−2^ cm^2^ V^−1^ s^−1^ (expect 1) magnitude and good on/off current ratio with minimized threshold voltage. Compounds 1 and 5 have resulted in ambipolar behaviour may be due to the substitutions (TAA and phenyl extended TAA) with a balanced hole and electron transport, which is very crucial in complementary circuits.^18^ Surprisingly, compound 3 has exhibited the highest hole mobility of 1.1 cm^2^ V^−1^ s^−1^ with good on/off ratio of 10^6^. In addition the threshold voltage is also observed as −2 V with no kink in the transfer curve. Good *I*_on/off_ ratio ensures minimum leakage and improved device performance. These values are ameliorated from many of the previous reports.^12^

**Table 4:** OFET characteristics of compounds 1 and 3–6

Comp. no	Channel	Mobility, *μ* (cm^2^ V^−1^ s^−1^)	*I*_on/off_	*V*_TH_ (V)
1	p	2.4 ×10^−2^	10^3^	−3
	n	1.5 ×10^−2^	10^4^	−2^a^
3	p	1.1	10^6^	−2
4	p	5.9 ×10^−2^	10^5^	−11
5	p	2.7 ×10^−2^	10^3^	−3
	n	13 ×10^−2^	10^4^	−1^a^
6	P	5.6 ×10^−2^	10^4^	−1

a Mobility calculated considering the kink observed in transfer curve.^17^

The methoxy group present in the TAA alter the electron distribution in the triarylamine system. It can assist the molecular self-assembly through pi–pi stacking and improve the communication among the molecules. From cyclic voltammetry analysis, it is observed that among the compounds studied, compound 3 has the lowest band gap (2.954 eV). In compounds 3, 4, and 6, although the phenanthroline moiety is clearly electron deficient, n-channel behaviour was not observed.^1,2^ Compounds 4 and 6 (thiophene and *t*-butyl) possess same HOMO levels at −5.6 eV and exhibited almost similar hole mobilities up to 5.9 and 5.6 × 10^−2^ cm^2^ V^−1^ s^−1^, respectively. In compound 3, the methoxy substitution may help the molecule to self-assemble in an efficient way to improve the communication among the molecules and resulted in highest mobility.

## Conclusions

In summary, a set of phenanthroline-TAAs has been designed, synthesized and investigated for their OFET behaviour. The compounds possessed HOMO levels in the range of −5.2 to −5.6 eV ensures less energy barrier for charge injection with thermal stability up to 560 °C. Solution processing from a chloroform solution has produced uniform film with high coverage area and good crystallinity as witnessed from SEM and thin film XRD analysis. The bottom gate top contact devices exhibited ambipolar behaviour with mobility of 10^−2^ cm^2^ V^−1^ s^−1^ and *I*_on/off_ 10^3^/10^4^. Especially, compound 3 with methoxyphenyl substitution in TAA side had resulted in p-channel behaviour with the highest mobility up to 1.1 cm^2^ V^−1^ s^−1^ and good *I*_on/off_ of 10^6^. This investigation gives an insight about effect of substituents in tuning the electronic behaviour of TAAs in OFET application.

## Conflicts of interest

There are no conflicts to declare.

## Supplementary Material

RA-010-D0RA00210K-s001
