# Supplementary material for: High performance p-channel and ambipolar OFETs based on imidazo[4,5-f]-1,10-phenanthroline-triarylamines
Source: RSC Adv. 2020 Mar 31;10(22):13043–9. doi: 10.1039/d0ra00210k (PMC9122573; doi:10.1039/d0ra00210k)
Supplement: RA-010-D0RA00210K-s001 [file RA-010-D0RA00210K-s001.pdf]

Electronic Supplementary Material (ESI) for RSC Advances.
This journal is © The Royal Society of Chemistry 2020

High performance *p*-channel and ambipolar OFETs based on Imidazo[4,5-f][1,10]phenanthroline-triarylamines

Ramachandran Dheepika,[a] Ramakrishnan Abhijnakrishna,[a] Predhanekar Mohamed Imran[b] and Samuthira Nagarajan[a]*

Table of Content | Page No.

Figure S1	Schematic representation of bottom gate top contact architecture OFET device	S3
Figure S2	Generalised representation of phenanthroline functionalized TAA	S4
Table S1	Geometrical parameters and Mullikan charges of compounds 1-6 (DFT:B3LYP (6-31g*)) at ground state	S4
Table S2	Energy, and mobility of compounds **1-6**	S4
Figure S3	Density of states graphs of compounds **1-6**. Inset is the corresponding optimized geometry.	S5
Table S3	Single crystal parameters of compounds **1-6**	S56
Figure S4	Frontier molecular orbitals (FMOs) distribution of compounds **1-6**	S7
Table S4	Fermi energy and interactions of compounds **1-6**	S7
Figure S5	Poly crystalline packing of compounds **1-6**	S8
Table S5	The electronic absorption behaviour of compounds **1-6**	S9
Table S6	Comparison of bond angle, bond distance and dihedral angle in ground state (GS) and excited state (ES)	S10
Figure S6	^{1}H and ^{13}C NMR spectra of compound **1**	S11
Figure S7	HR-mass spectrum of compound **1**	S12
Figure S8	^{1}H and ^{13}C NMR spectra of compound **2**	S13
Figure S9	HR-mass spectrum of compound **2**	S13
Figure S10	^{1}H and ^{13}C NMR spectra of compound **3**	S14
Figure S11	HR-mass spectrum of compound **3**	S15
Figure S12	^{1}H and ^{13}C NMR spectra of compound **4**	S16
Figure S13	HR-mass spectrum of compound **4**	S16
Figure S14	^{1}H and ^{13}C NMR spectra of compound **5**	S17
Figure S15	HR-mass spectrum of compound **5**	S18
Figure S16	^{1}H and ^{13}C NMR spectra of compound **6**	S19
Figure S17	HR-mass spectrum of compound **6**	S19

Spectral data

4-(1H-imidazo[4,5-f][1,10]phenanthrolin-2-yl)-N,N-diphenylaniline, **1**:

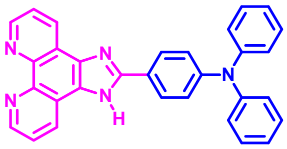

Yellow solid. Yield: 98 %. M.p: 343 °C. H 400 MHz (δ, ppm): 13.640 (s, 1H), 9.030 (d, J=2Hz, 2H), 8.914 (d, J=7.6Hz, 2H), 8.167 (d. J=8Hz, 2H), 7.387 (d, J=7.6Hz, 4H), 7.137 (d, J=6Hz, 10H). ^{13}C 100MHz (δ, ppm): 151.09, 148.98, 148.06, 147.15, 143.74, 130.22, 130.09, 127.96, 125.32, 125.25, 124.34, 123.81, 123.71, 122.44. HRMS (ESI) m/z calcd for $C_{31}H_{21}N_5$ + H [M+H] 464.1875 found 464.1891.

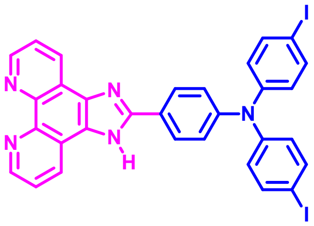

4-(1H-imidazo[4,5-f][1,10]phenanthrolin-2-yl)-N,N-(di(4-iodophenyl))aniline **2**:

Dark yellow solid. Yield: 98 %. M.p: 330 °C ^{1}H 400 MHz (δ, ppm): 13.144 (s, 1H), 9.853 (d, J=9.2Hz, 1H), 9.111-9.047, (m, 2H), 8.893, (d, J=6.8Hz. 1H), 8.193 (d, J=6.4Hz, 2H), 7.945 (d, J=6Hz, 1H), 7.454 (s, 8H), 7.266-7.247 (m, 6H), 6.992 (d, J=9.6Hz, 4H), 3.865 (s, 6H) ^{13}C 100 MHz (δ, ppm): 158.86, 152.48, 151.18, 148.69, 147.75, 145.68, 143.95, 135.79, 133.23, 132.77, 131.83, 128.52, 128.40, 127.59, 127.39, 126.71, 125.00, 124.05, 123.89, 122.65, 114.15, 55.24. HRMS (ESI) m/z calcd for $C_{31}H_{19}N_5I_2$ + H [M+H] 715.9808 found 715.9834.

4-(1H-imidazo[4,5-f][1,10]phenanthrolin-2-yl)-N,N-(di(4-methoxyphenyl)phenyl)aniline, **3**:

Pale yellow solid. Yield: 96 %. M.p: 373 °C ^{1}H 400 MHz (δ, ppm): 13.234 (s, 1H), 9.792 (d, J=8Hz, 1H), 9.343 (s, 1H), 9.101-

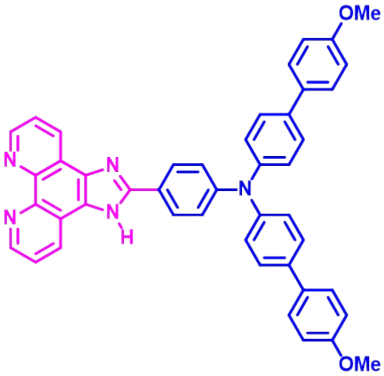

9.034 (m, 2H), 8.908 (d, J=7.6Hz, 1H), 8.196 (d, J=8.4Hz, 2H), 7.931 (s, 1H), 7.708 (s, 1H), 7.591-7.571 (m, 5H), 7.476-7.422 (m, 5H), 7.277-7.216 (m, 5H) ^{13}C 100 MHz (δ, ppm): 152.60, 151.17, 148.58, 145.98, 144.06, 141.56, 133.49, 131.16, 127.74, 127.33, 126.36, 126.03, 124.97, 124.25, 124.17, 122.93, 119.67. HRMS (ESI) m/z calcd for $C_{45}H_{33}N_5O_2$ + H [M+H] 676.2716 found 676.2736.

4-(1H-imidazo[4,5-f][1,10]phenanthrolin-2-yl)-N,N-(di(4- thiophen-3-yl)phenyl)aniline, 4:

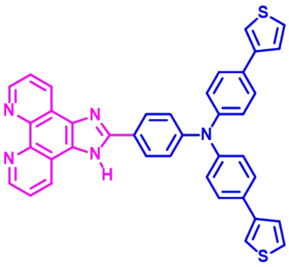

Yellow solid. Yield: 95 %. M.p: 295 °C. ^{1}H 400 MHz (δ, ppm): 13.304 (s, 1H), 9.098-8.934 (m, 4H), 8.216 (d, J=8Hz, 2H), 7.719 (t, J=6.4Hz, 2H), 7.643-7577 (m, 8H), 7.452 (t, J=6.8Hz, 4H), 7.354-7.278 (m, 8H) ^{13}C 100 MHz (δ, ppm): 155.98, 153.38, 152.62, 151.09, 148.70, 144.98, 140.83, 134.83, 133.63, 132.51, 131.86, 131.37, 129.76, 129.06, 127.86, 127.72. HRMS (ESI) m/z calcd for $C_{39}H_{25}N_5S_2$ + H [M+H] 628.1630, found 628.1656.

(4-(1H-imidazo[4,5-f][1,10]phenanthrolin-2-yl)-N,N-(di(biphenyl))aniline, 5:

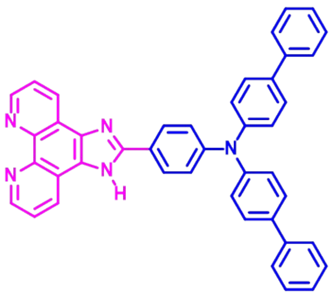

Yellow solid. Yield: 97 %. M.p: 376 °C. ^{1}H 400 MHz (δ, ppm): 8.863-8.713 (m, 4H), 8.145 (d, J=7.6Hz, 2H), 7.475-7.264 (m, 6H) 6.913 (d, J=8.0Hz, 2H) 6.736 (d, J=8.0Hz, 4H) ^{13}C 100MHz (δ, ppm): 151.64, 147.78, 147.36, 146.39, 143.69, 138.89, 138.45, 138.09, 130.60, 128.01, 127.67, 126.44, 124.76, 124.20, 123.24, 86.92. HRMS (ESI) m/z calcd for $C_{43}H_{29}N_5$ + H [M+H] 616.2501, found 616.2456.

(4-(1H-imidazo[4,5-f][1,10]phenanthrolin-2-yl) N,N-(di(4-(tert-butyl)-biphenyl)aniline, **6**:

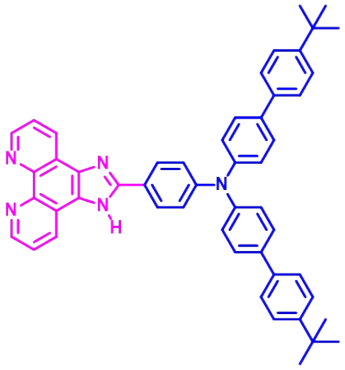

Yellow solid. Yield: 96 %. M.p: 356°C. ^{1}H 400 MHz (δ, ppm): 8.958-8.734 (m, 4H), 8.133-8.042 (m, 2H), 7.895 (d, J=8.6Hz, 2H), 7.529-7.353 (m, 10H), 7.198-7.043 (m, 9H) 1.773 (s, 1H), 1.321 (s, 18H) ^{13}C 100MHz (δ, ppm): 151.86, 151.21, 146.79, 132.83, 132.72, 131.29, 131.25, 130.40, 129.05, 127.81, 125.70, 125.38 , 124.22, 124.17, 123.89, 123.04, 120.266, 35.02, 31.29. HRMS (ESI) m/z calcd for $C_{51}H_{46}N_5$ + H [M+H] 729.2753, found 729.2955

Device architecture

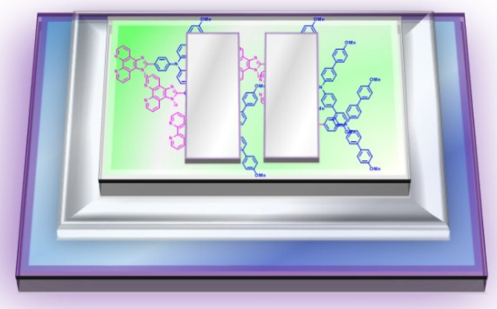

Figure S1. Schematic representation of bottom gate top contact architecture OFET device

Computational basis

To understand the theoretical aspects of the phenanthroline imidazole functionalized TAAs density functional theory (DFT) has been applied. The optimized geometry, crystalline parameters, density of states, and polycrystalline packing at ground state are investigated. The investigation is extended to time dependant DFT to scrutinise the excited state geometrical behaviour of the molecules. The frontier molecular orbitals (FMOs) are qualitatively represented in Figure S4. HOMO is spread mainly over the TAA side of the molecule and LUMO is distributed over the phenanthroline side including the azole ring. DOS graphs has given an idea about the electron distribution and the available levels for charge carrier movement.

Geometrical parameters of these new molecules are elucidated using DFT:B3LYP (*ab-initio*) approach. The molecules were optimised by Gaussian DFT:B3LYP (6-31g*) in ground state. To find the available Fermi levels density of states energy is computed by PBE functional and visualised as graphs in Figure S3.

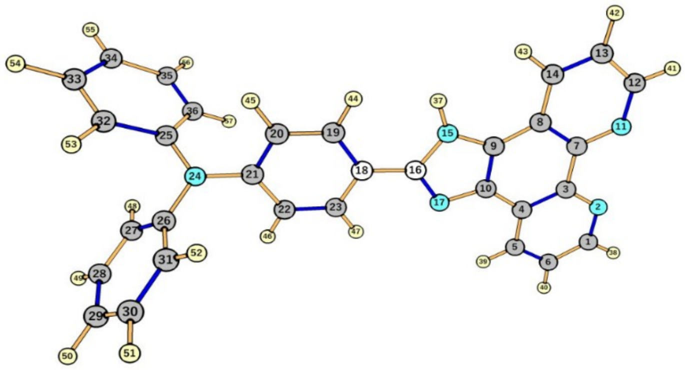

Figure S2. Generalised demonstration of phenanthroline functionalized TAA

Table S1. Geometrical parameters and Mulliken charges of compounds **1**-**6** (DFT:B3LYP (6-31g*)) at ground state

Comp. No	Charge				Bond length (Å)			Dihedral angle		Dipole moment
	C_{16}	N_{15}	N_{24}	C_{21}	NC_{16}	NC_{21}	CC_{18}	(21-24-25-26)	(15-16-18-19)	(Debye)
**1**	0.384	-0.833	-0.820	0.298	1.394	1.419	1.456	0.034	3.95	6.99
**2**	0.023	-0.040	-0.350	0.330	1.345	1.430	1.463	0.098	3.76	4.99
**3**	0.384	-0.832	-0.826	0.298	1.394	1.419	1.455	0.232	6.27	7.37
**4**	0.384	-0.832	-0.825	0.294	1.394	1.421	1.456	0.211	5.69	6.29
**5**	0.384	-0.826	-1.833	0.295	1.394	1.420	1.456	0.192	5.74	6.78
**6**	0.384	-0.826	-0.832	0.296	1.395	1.420	1.455	0.224	6.64	7.44

Table S2. Energy, and mobility of compounds **1**-**6**

Comp. No	Vol (Å)^3	Density (Mg/m^3)	Energy (eV)	Band energy Initial- Final DOSPROCAR	DOS Gap (eV)	Mobility x 10^{-4} (cm^2/v/s)
**1**	2330.46	0.330	-398.82	-24.33/2.32	-2.050	3.359
**2**	2586.03	0.459	-393.71	-24.19/2.11	-2.034	2.878
**3**	1633.89	0.069	-582.74	-26.24/0.71	-2.052	0.328
**4**	3617.75	0.288	-493.28	-24.50/1.82	-2.030	0.403
**5**	3806.74	0.269	-537.29	-24.55/1.87	-2.204	3.912
**6**	5236.31	0.231	-670.45	-24.79/2.02	-2.060	4.232

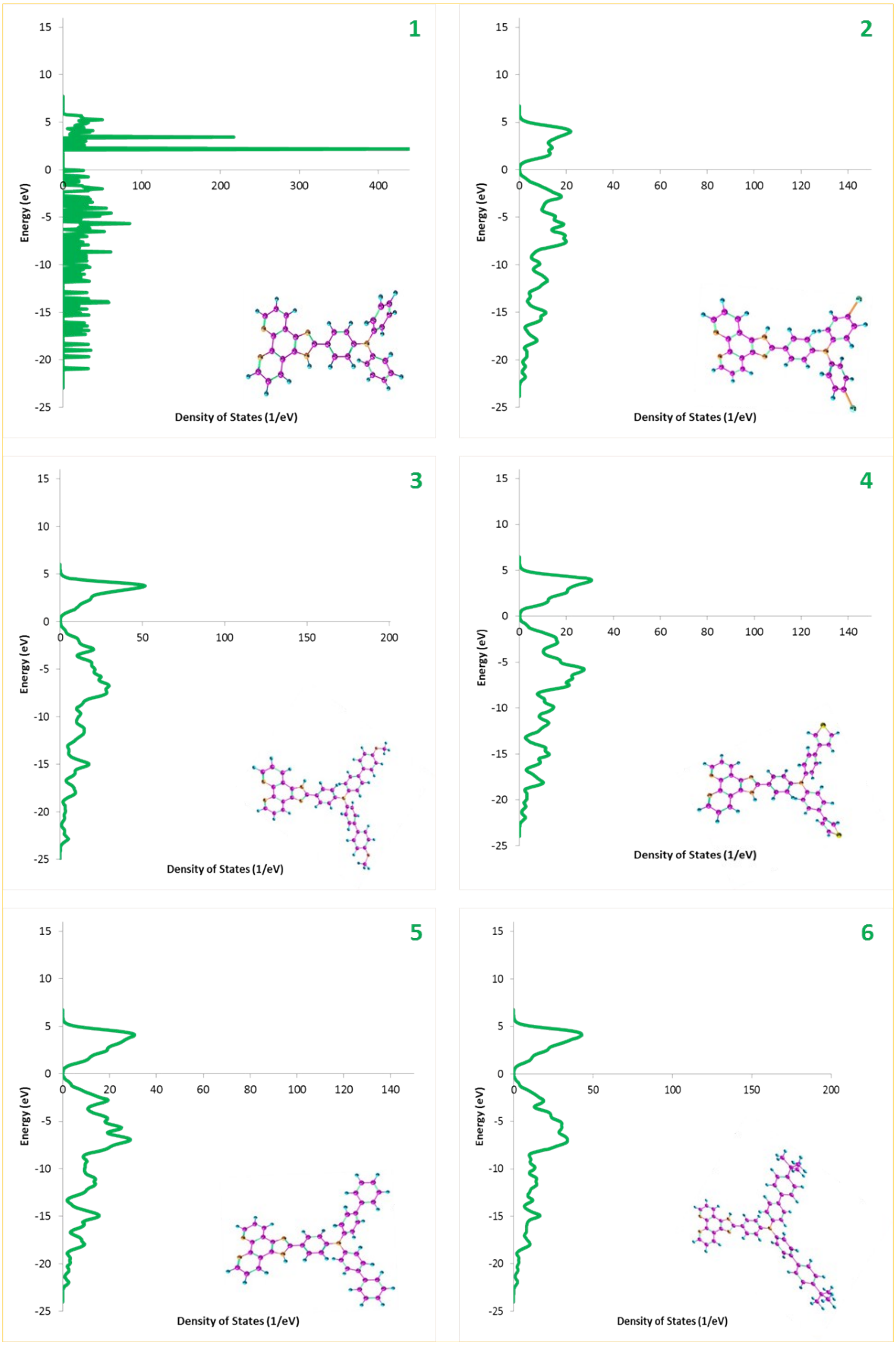

Figure S3. Density of states graphs of compounds **1**-**6**. Inset is the corresponding optimized geometry.

The precise single crystal parameters were achieved from MedeA using these optimised structures obtained by DFT:B3LYP. The poly crystalline packing modes of the molecules were obtained through an incremental fashion by Discovery Studio Viewer. Many space groups from Cambridge structural database were tried sequentially to monitor the interactive distance and packing pattern. From the single crystal predictions volume, density, energy values are calculated and given in Table S3. The compounds are predicted in orthorhombic system (compound 2 in cubic, due to the presence of iodine atom) and the crystalline parameters are given in Table S4.

Table S3. Single crystal parameters of compounds **1**-**6**

Compd. No.	Type	Sides			Angles		
		a	b	c	α	β	γ
**1**	Orthorhombic (P- 212121)	20.01	12.88	9.04	90.00	90.00	90.00
**2**	Cubic (P212121)	25.37	25.37	25.37	90.00	90.00	90.00
**3**	Orthorhombic (P4)	22.28	19.15	8.48	90.00	90.00	90.00
**4**	Orthorhombic (P2)	22.73	20.66	8.10	90.00	90.00	90.00
**5**	Orthorhombic (P21)	23.54	25.75	8.63	90.00	90.00	90.00
**6**	Orthorhombic (PCC2)	20.09	15.04	8.55	90.00	90.00	90.00

Frontier molecular orbital (FMOs) energies have been calculated and the electron distribution is visualised in Figure S4. In all the compounds, highest occupied molecular orbital (HOMO) is spread all over the molecule and the lowest unoccupied molecular orbital (LUMO) is found denser only over the phenanthroline side including the azole ring. This distribution clearly shows that the substitutions in TAA side have important role in tuning the energy levels. In compounds **3**, **4** and **6**, LUMO is not seen in the TAA side which restricts the electron flow and thus no *n*-channel behaviour is observed in OFET devices.

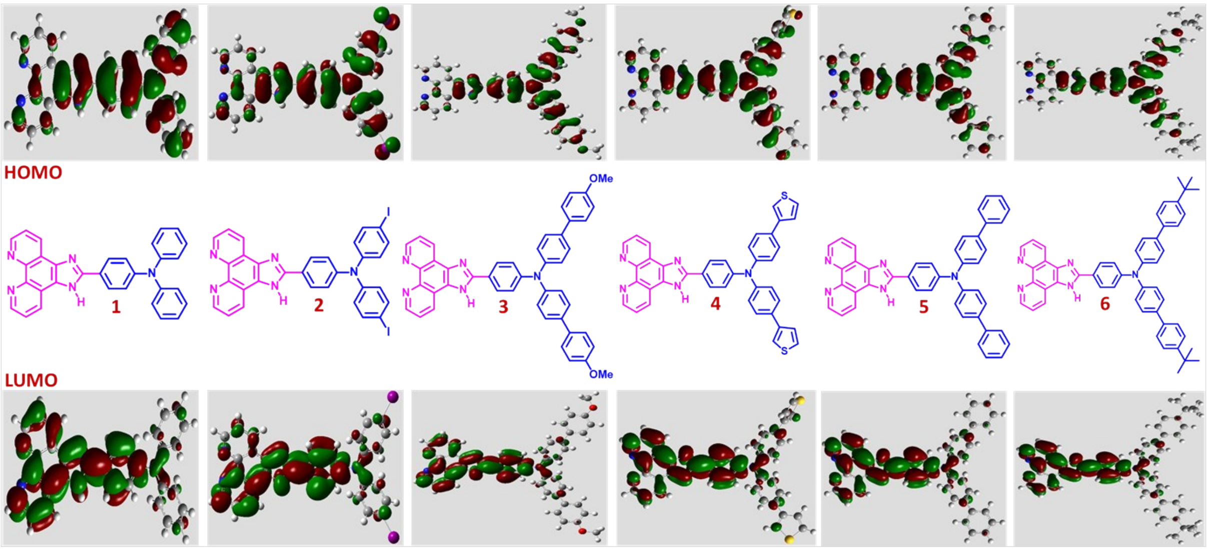

Figure S4. Frontier molecular orbitals (FMOs) distribution of compounds **1**-**6**

The DFT calculations were performed with the Vienna ab initio simulation package (VASP) at local density approximation (LDA) and generalized gradient approximation (GGA) levels of theory. Out of the available GGA functionals, we selected PBE functional where dispersion corrections were included according to D2 scheme. The PBE (pertaining to dispersion evaluation) calculations were performed to evaluate pi-pi interactions. However, as all the molecules are non-planar, building a dimer and optimising the desired sequence was difficult. They oriented in a hip-hop or zig-zag pattern and no significant information about pi-pi stacking was acquired. To compute Van der Waal's forces in a medium, solvent is treated as a continuum. Where, the polarizability, dielectric permittivity, orientation, and induction interaction energies of each molecule was considered. GGA-BLYP DFT-D2 was used to account for the Van der Waal's energy with respect to non-dispersive and Van der Waal's forces. Pair interactions value suggests that more non-covalent interactions are possible than pi-pi

interactions. No feasible distance was obtained from crystal lattice parameters for pi-pi interactions.[1] The Van der Waals radius was set to a standard 30 Å for pair interactions (Table S5).

Table S4. Fermi energy and interactions of compounds **1**-**6**

Comp. No	Fermi energy (PBE)	Electron density (10^{28} e^-/m^3)	vdW energy	VASP energy DFT-D2 (eV) PBE	Non-dispersive: Van der Waals DFT-D2 (eV)	Pair interactions (vdW) radius=30Å S6=1.2Å, D=20
1	-3.371	2.796	-1.518	-398.820<br>-384.237	-1.5185	80467
2	-3.359	1.666	-1.702	-393.719<br>-378.206	-1.7021	71416
3	-3.394	2.737	-2.264	-582.744<br>-562.501	-2.2643	25338
4	-3.511	2.839	-1.944	-493.283<br>-474.051	-1.9442	81169
5	-3.516	1.889	-2.108	-537.294<br>-518.399	-2.1084	90225
6	-3.581	2.422	-3.052	-670.458<br>-644.005	-3.0516	112465

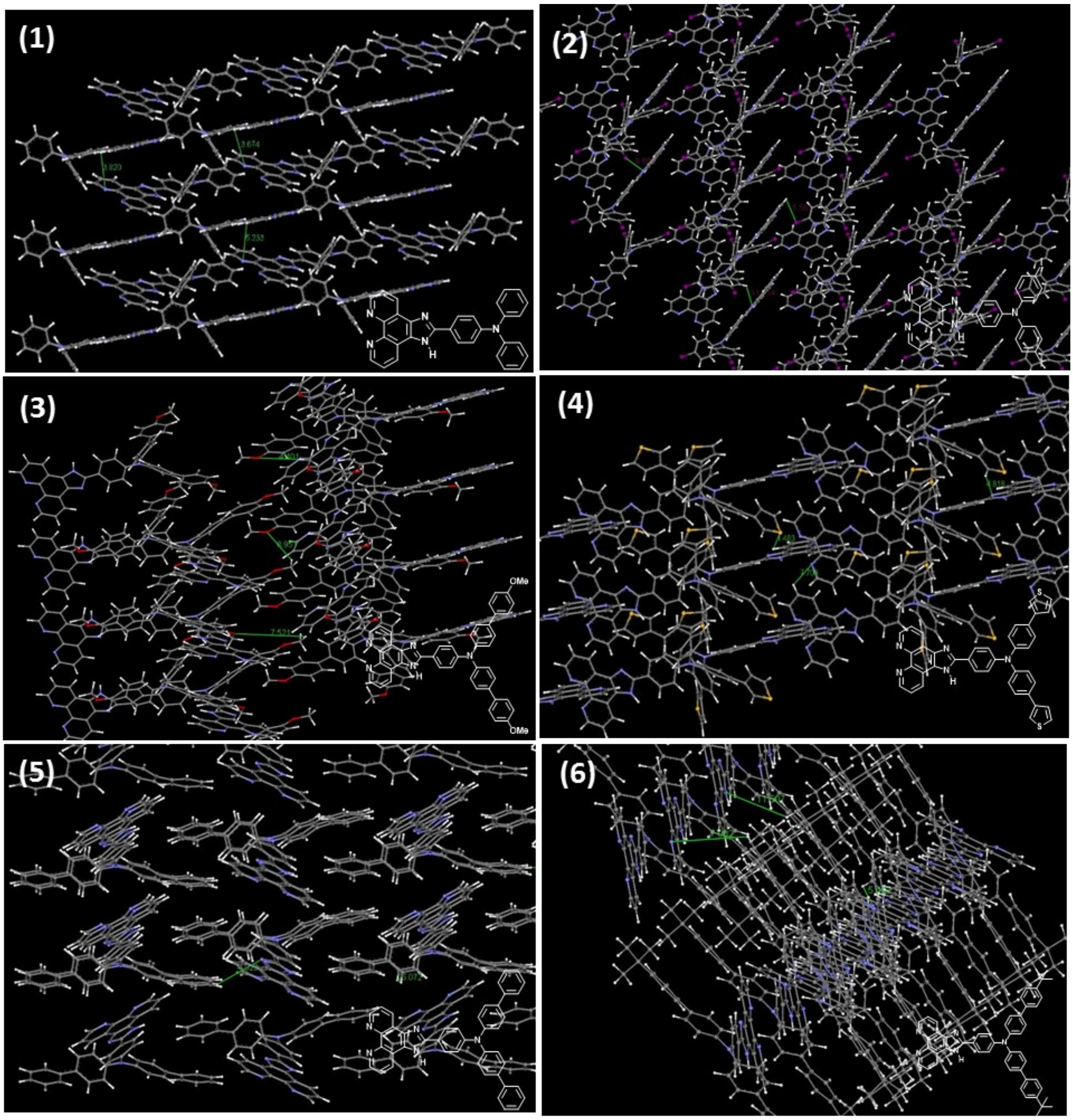

Figure S5. Poly crystalline packing of compounds **1**-**6**

The theoretical calculations using the TDDFT (time dependent-DFT) suggests significant spectral insights which are quite in agreement with the experimental calculations. The Table S7 shows the comparison of bond length, bond angle and dihedral angle in ground state (GS) and excited state (ES); out of the several values, only four values with good frequency factor have been chosen. A look at the geometrical parameters suggests that none of these systems are planar (Table S7). A slight change in the dihedral angle between GS and ES is observed which affects the delocalization of molecular orbitals. Especially, in compound **2** no change was observed in GS and ES probably due to the presence of iodine atoms. Iodine is a large atom and balances the changes in electronic clouds at molecular level. This molecule has intense absorption at 366 nm pertaining to a transition from GS singlet to an ES level four the LUMO and signifies the absorption is spread over a large molecular level.

From the oscillator strength value (F) obtained from the TD-DFT calculations, all the compounds have S_0-S_1 and S_0-S_3 or S_0-S_5 as the two possible transitions. Noticeably in all the cases S_0-S_1 is the prominent transition with an F value of 1.0 or 1.1 and transitions to triplet states are forbidden with respect to the F values. Except for compound **2**, the others have S_0-S_3 as the second probable transition. On comparing the absorption wavelength with the experimental values a very close agreement was obtained. For compounds **1**, **3**-**6** S_0-S_3 transitions are prominently observed and exceptionally for compound **2** the transition is pronounced in S_0-S_5 states due to the heavy atom.

Table S5. The electronic absorption behaviour of compounds **1**-**6**

Compound	Wavelength (nm)	Energy (eV)	F	Type of transition
**1**	517.5	2.395	0.000	S_0-T_1
	392.1	3.162	1.013	S_0-S_1
	360.9	3.434	0.063	S_0-S_3
	339.6	3.650	0.131	S_0-S_5
**2**	507.9	2.441	0.000	S_0-T_1
	393.8	3.148	0.000	S_0-T_2
	387.6	3.198	1.044	S_0-S_1
	351.6	3.526	0.225	S_0-S_5
**3**	527.3	2.351	0.000	S_0-T_1
	426.4	2.907	0.000	S_{-1}-S_1
	415.3	2.985	1.067	S_0-S_1
	367.7	3.372	0.545	S_0-S_3
**4**	525.8	2.358	0.000	S_0-T_1
	416.5	2.977	0.000	S_0-T_2
	410.8	3.018	1.068	S_0-S_1
	374.6	3.309	0.436	S_0-S_3
**5**	524.3	2.364	0.000	S_0-T_1
	421.7	2.940	0.000	S_0-T_2
	409.4	3.028	1.109	S_0-S_1
	371.7	3.335	0.485	S_0-S_3
**6**	525.8	2.357	0.000	S_0-T_1
	424.2	2.922	0.000	S_0-T_2
	412.6	3.005	1.211	S_0-S_1
	370.5	3.346	0.597	S_0-S_3

Dihedral angle (Figure S2 and Table S1) of the central nitrogen atom of TAA and the azole nitrogen are calculated. Compounds **1** and **2** has the lower dihedral angle may be due to relatively less pi- conjugation. Similarly, compounds **3** and **6** with extended methoxy and t-butyl phenyl substitution have resulted in 6.3 and 6.6 dihedral angle and compounds **4** and **5** has moderate values. This trend is observed in both central nitrogen atoms. In terms of dipole moments, all the compounds (Except **2**) have good dipole moment value ranging from 6.3 to 7.0 debye. In compound **2**, due to the presence of heavy atom, iodine, the value is comparatively low at 4.99 debye among the studied molecules.

Table S6. Comparison of bond angle, bond distance and dihedral angle in ground state (GS) and excited state (ES)

Comp. No	Bond distance (Å)			Bond angle (°) ($A_{15\text{-}16\text{-}18}$)		Dihedral angle (°) (15-16-18-19)		Dihedral angle (°) (21-24-25-26)	
	Bond labels	G.S.	E. S.	G. S	E. S	G. S	E. S	G. S	E. S
**1**	$d_{16\text{-}18}$<br>$d_{21\text{-}24}$	1.456<br>1.419	1.459<br>1.429	124.37	126.21	1.869	3.958	0.034	0.035
**2**	$d_{16\text{-}18}$<br>$d_{21\text{-}24}$	1.463<br>1.430	1.463<br>1.432	125.23	125.23	1.884	1.884	0.098	0.099
**3**	$d_{16\text{-}18}$<br>$d_{21\text{-}24}$	1.455<br>1.418	1.455<br>1.483	125.19	124.26	3.018	2.960	0.232	0.237
**4**	$d_{16\text{-}18}$<br>$d_{21\text{-}24}$	1.456<br>1.421	1.458<br>1.431	124.32	124.32	2.689	5.693	0.211	0.211
**5**	$d_{16\text{-}18}$<br>$d_{21\text{-}24}$	1.457<br>1.421	1.457<br>1.432	127.19	124.29	4.851	2.714	0.192	0.192
**6**	$d_{16\text{-}18}$<br>$d_{21\text{-}24}$	1.455<br>1.420	1.455<br>1.422	125.19	126.17	3.199	5.594	0.224	0.228

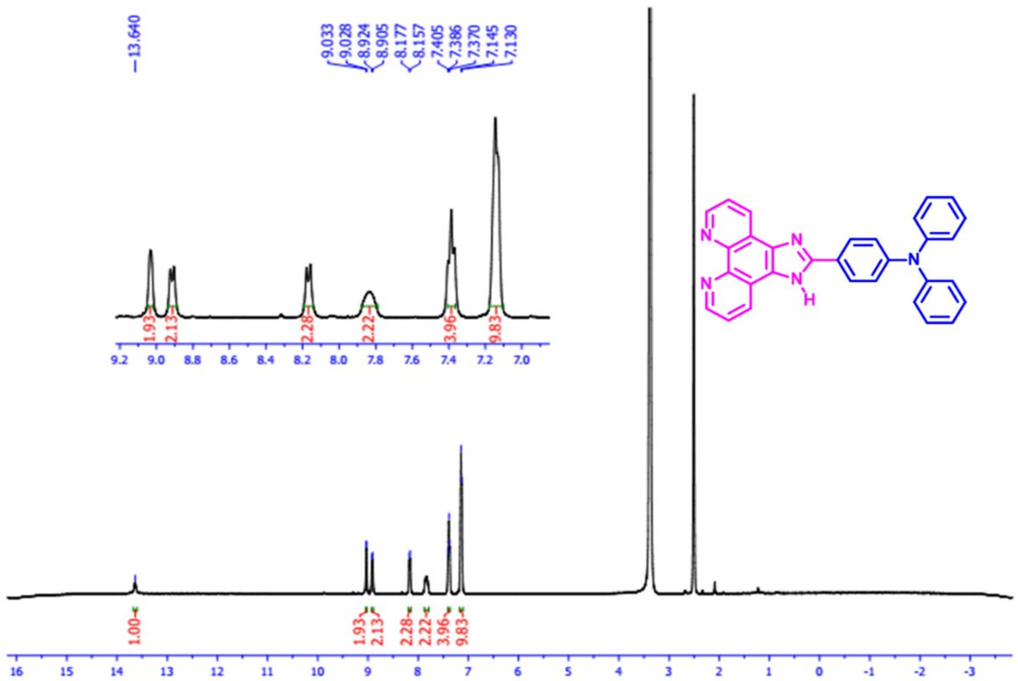

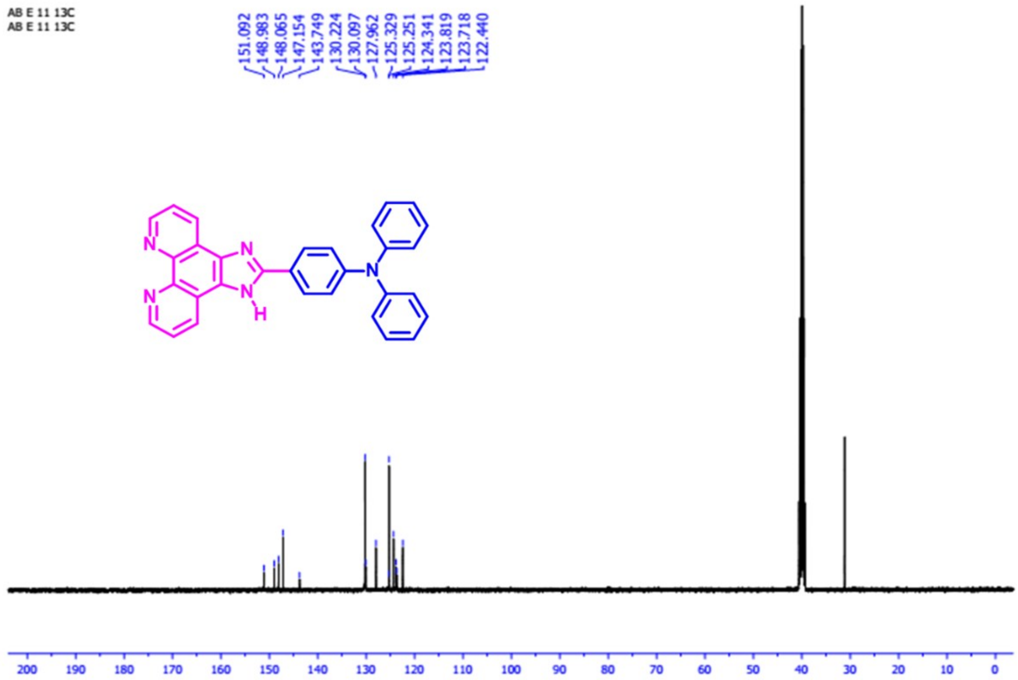

Figure S6. ^{1}H and ^{13}C NMR spectra of compound **1**

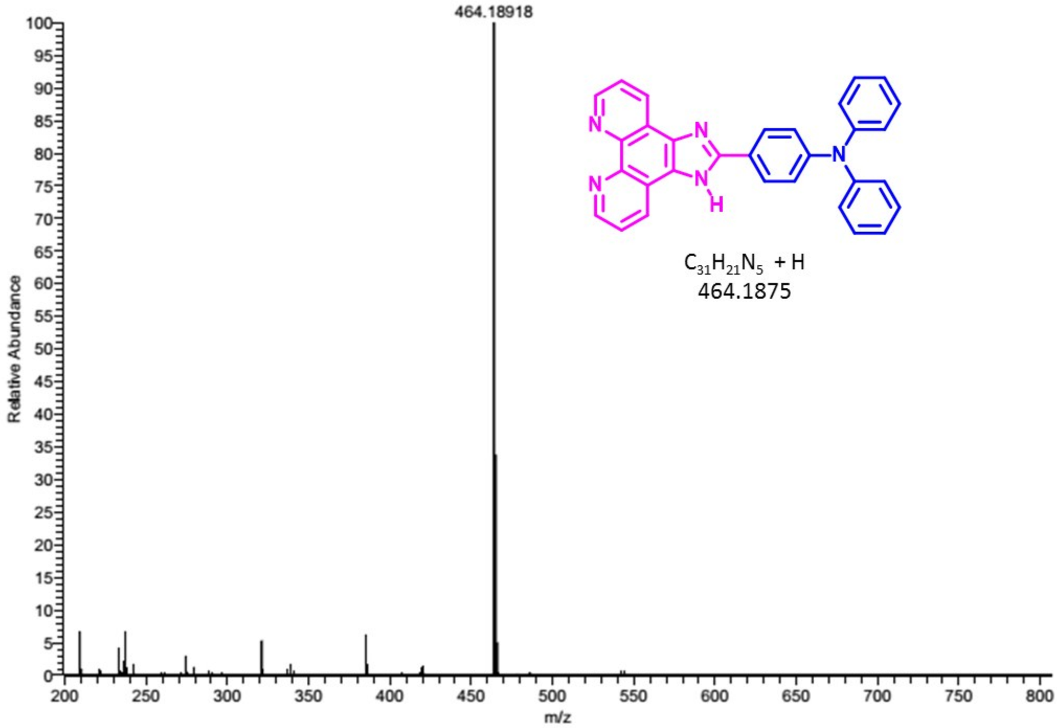

Figure S7. HR-mass spectrum of compound **1**

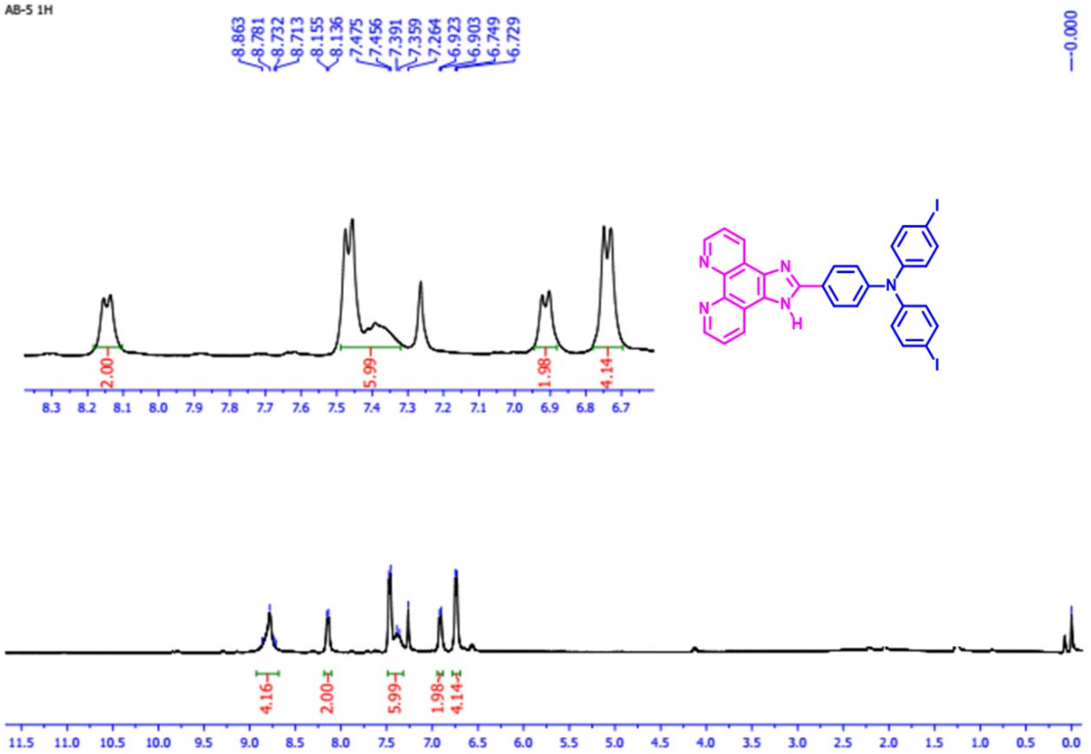

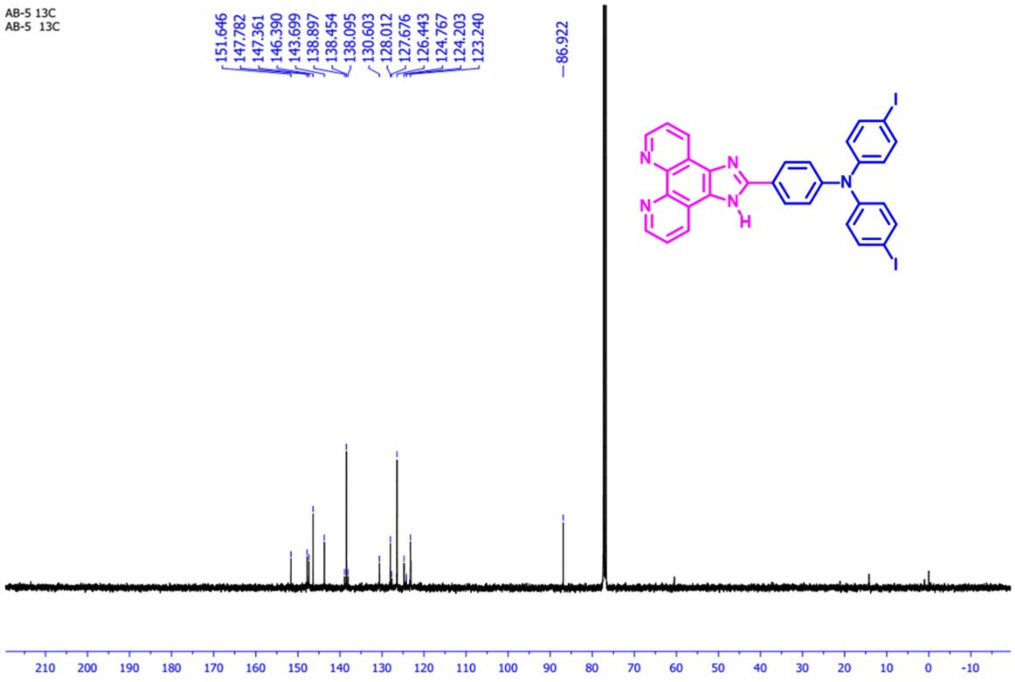

Figure S8. ^{1}H and ^{13}C NMR spectra of compound **2**

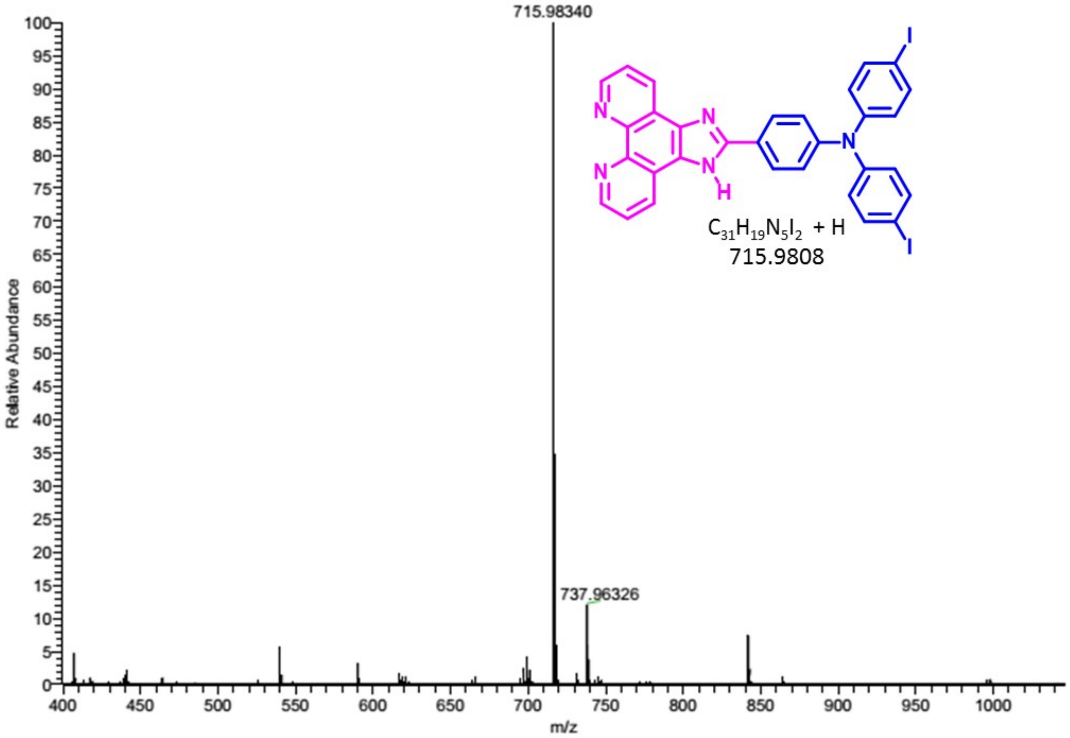

Figure S9. HR-mass spectrum of compound **2**

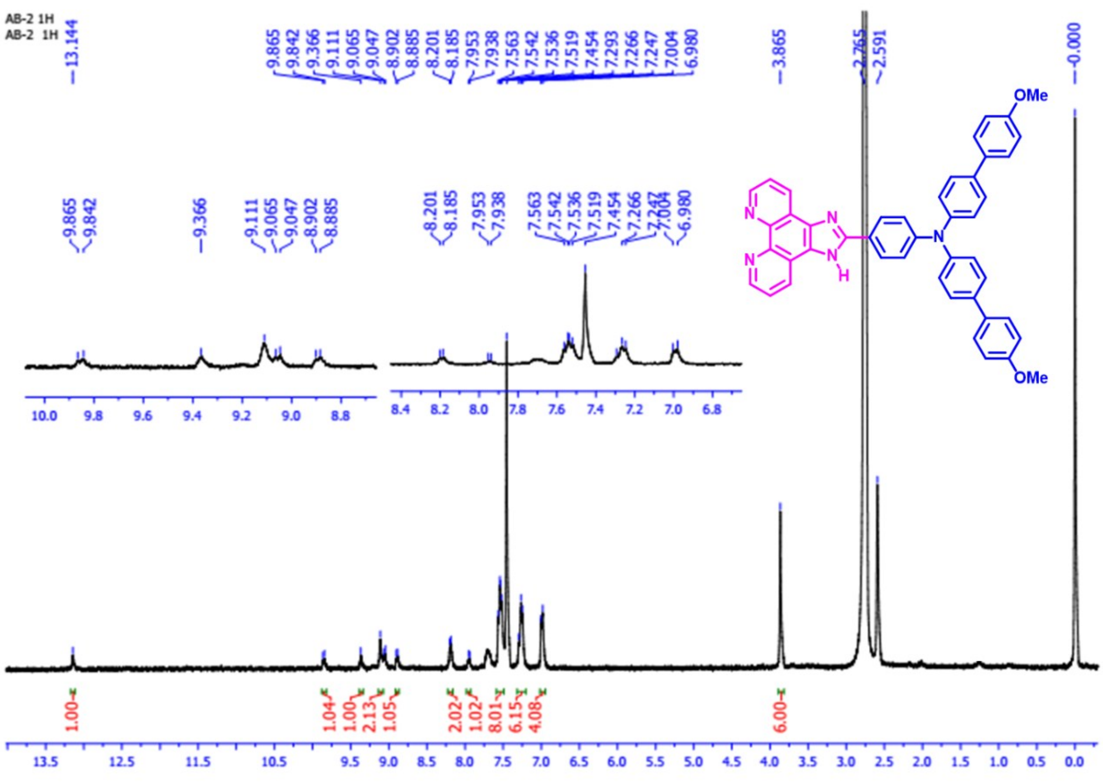

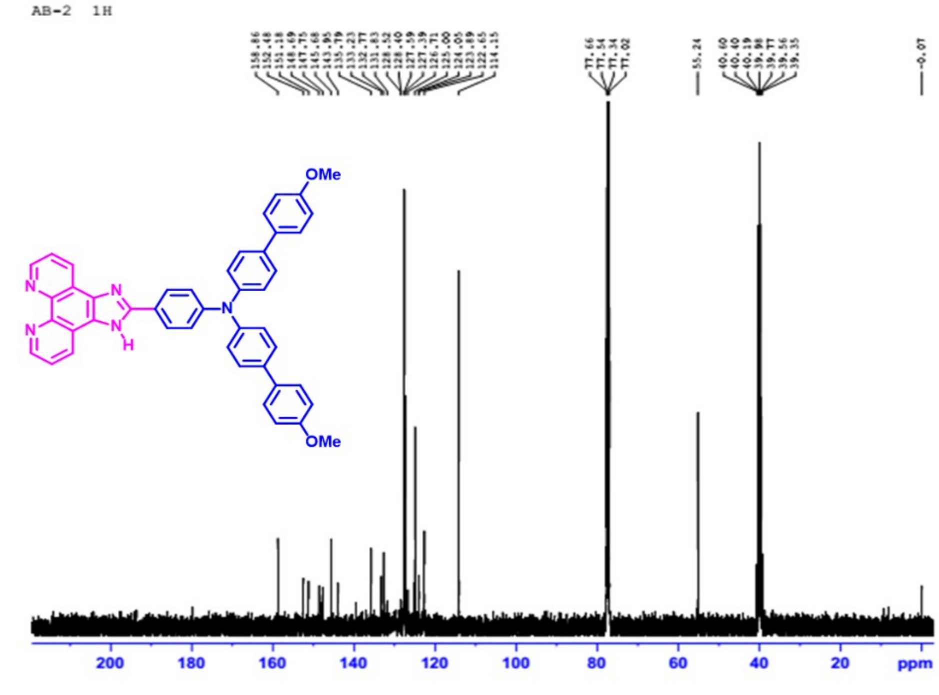

Figure S10. ^{1}H and ^{13}C NMR spectra of compound **3**

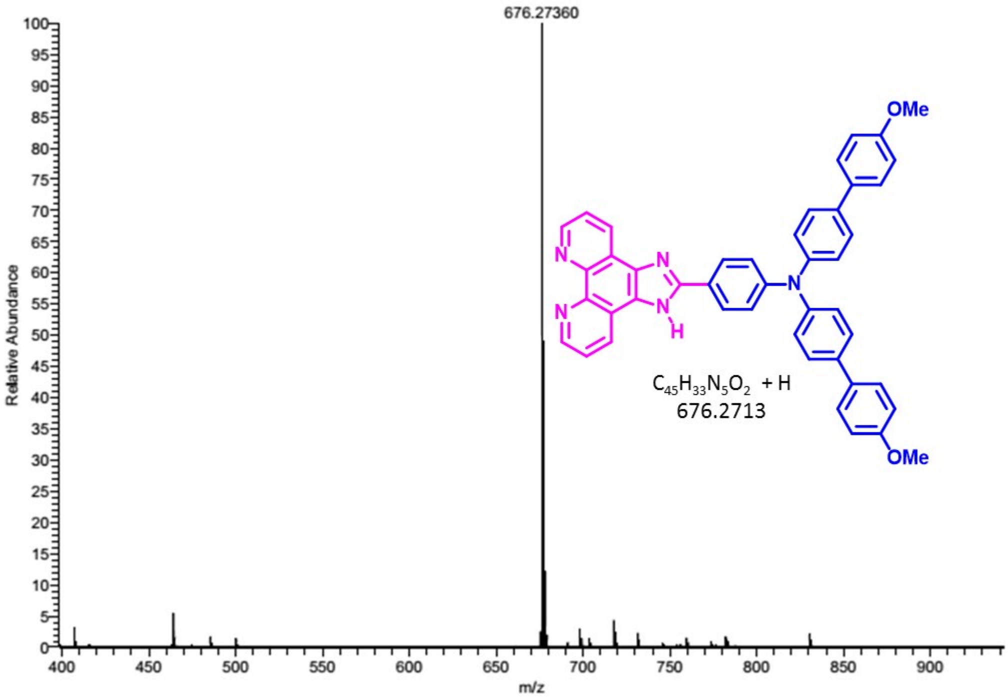

Figure S11. HR-mass spectrum of compound **3**

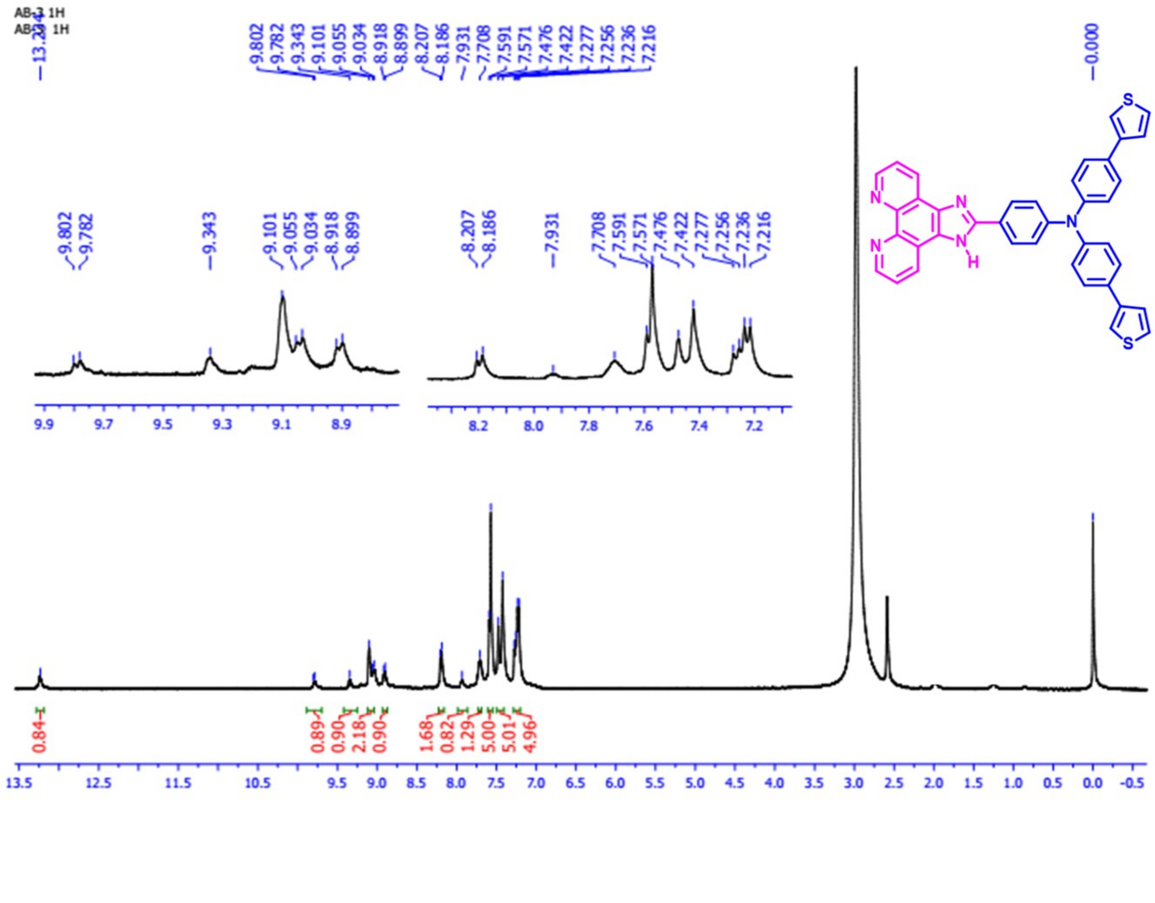

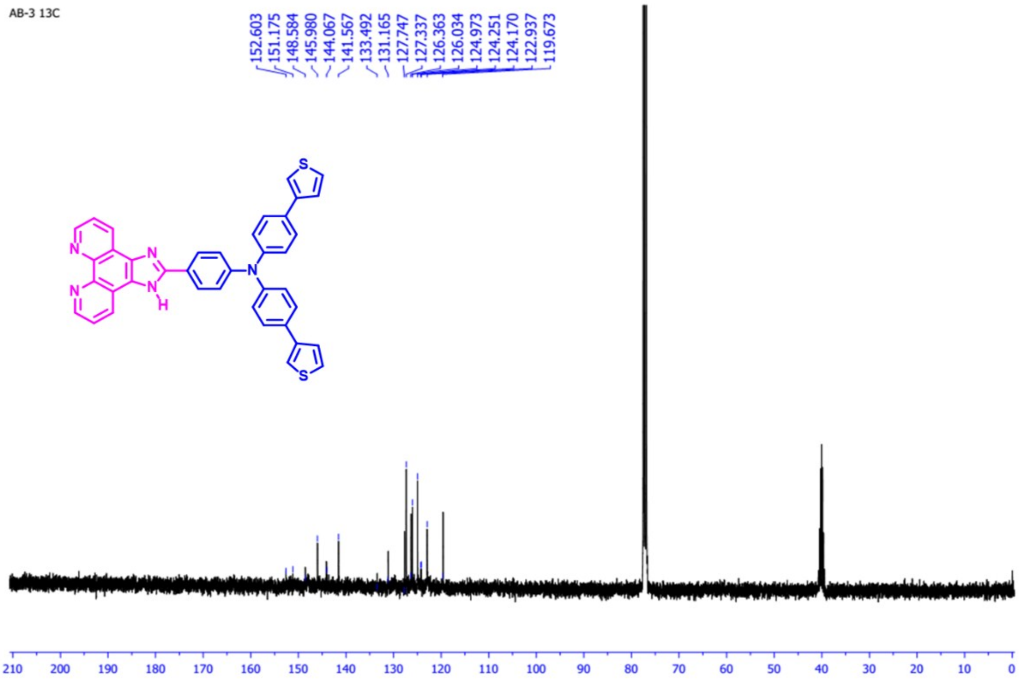

Figure S12. ^{1}H and ^{13}C NMR spectra of compound **4**

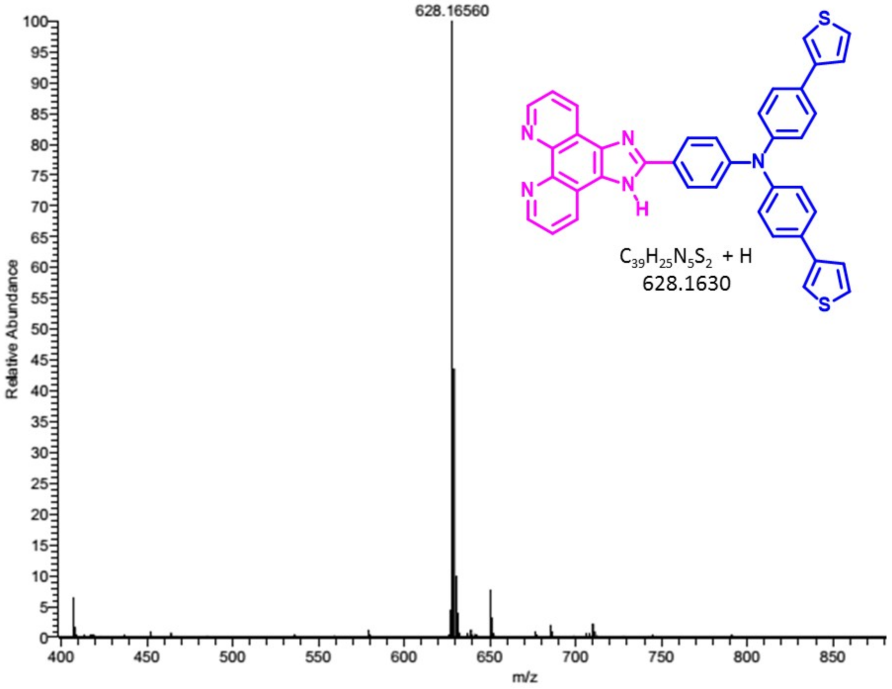

Figure S13. HR-mass spectrum of compound **4**

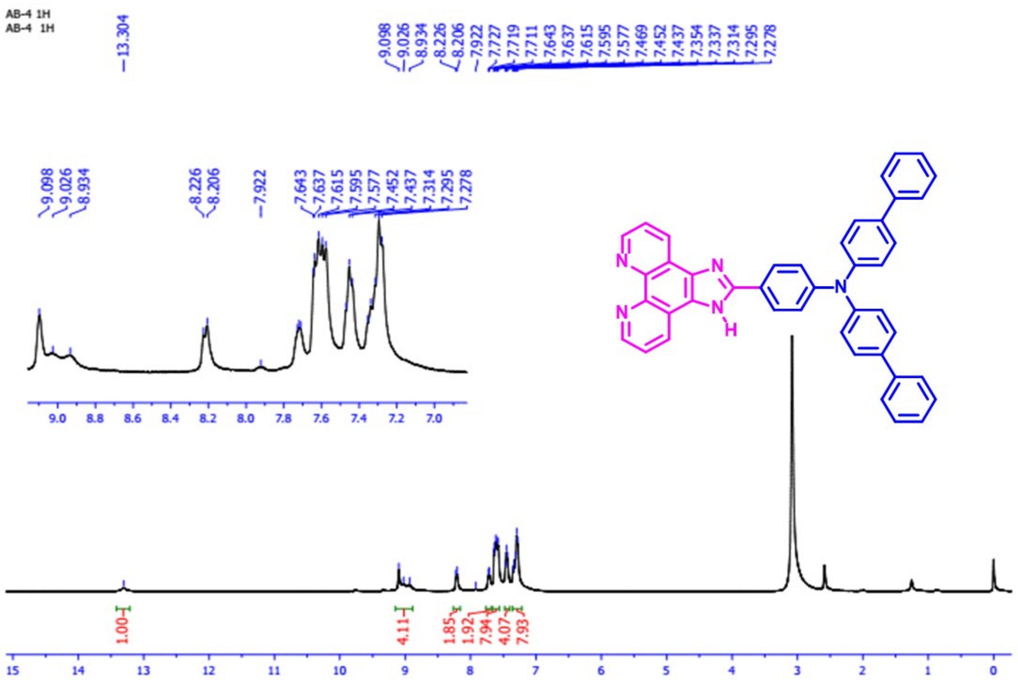

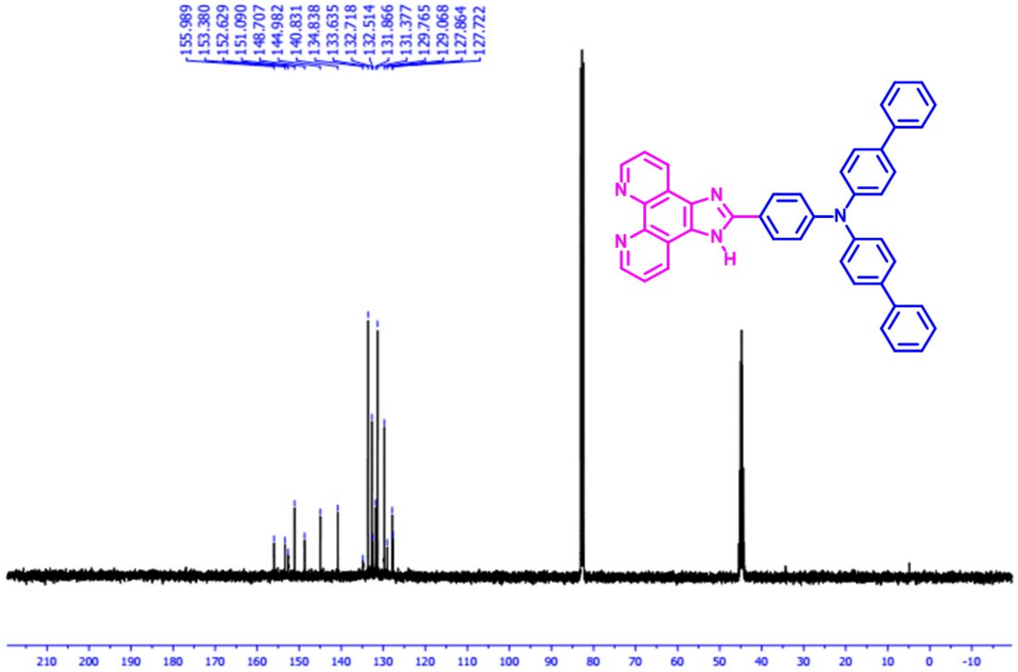

Figure S14. ^{1}H and ^{13}C NMR spectra of compound **5**

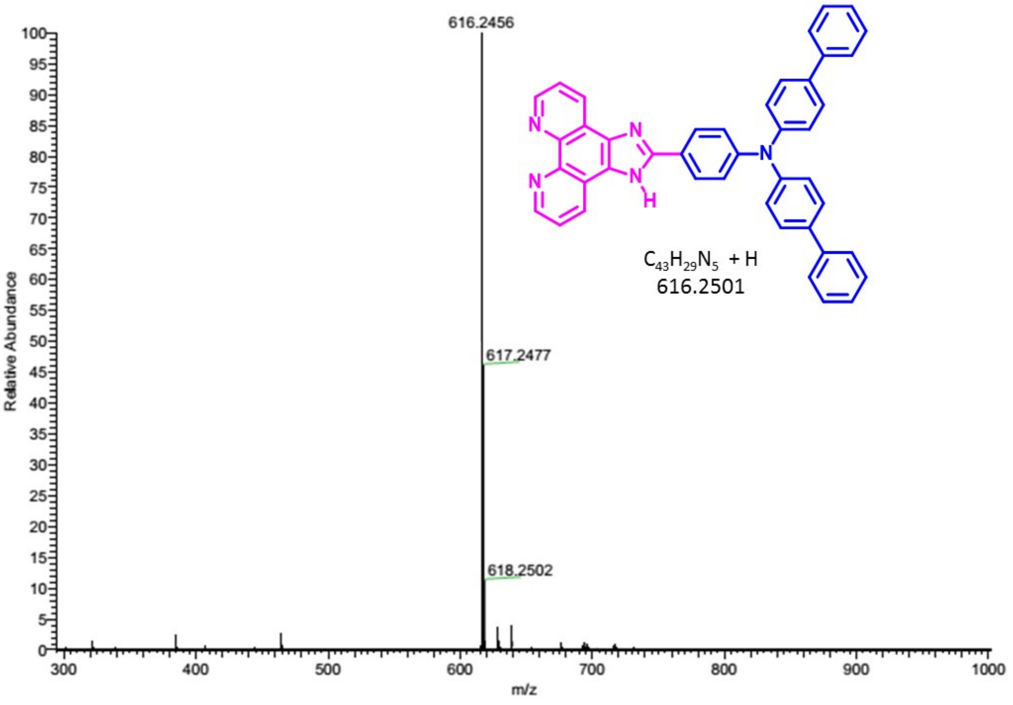

Figure S15. HR-mass spectrum of compound **5**

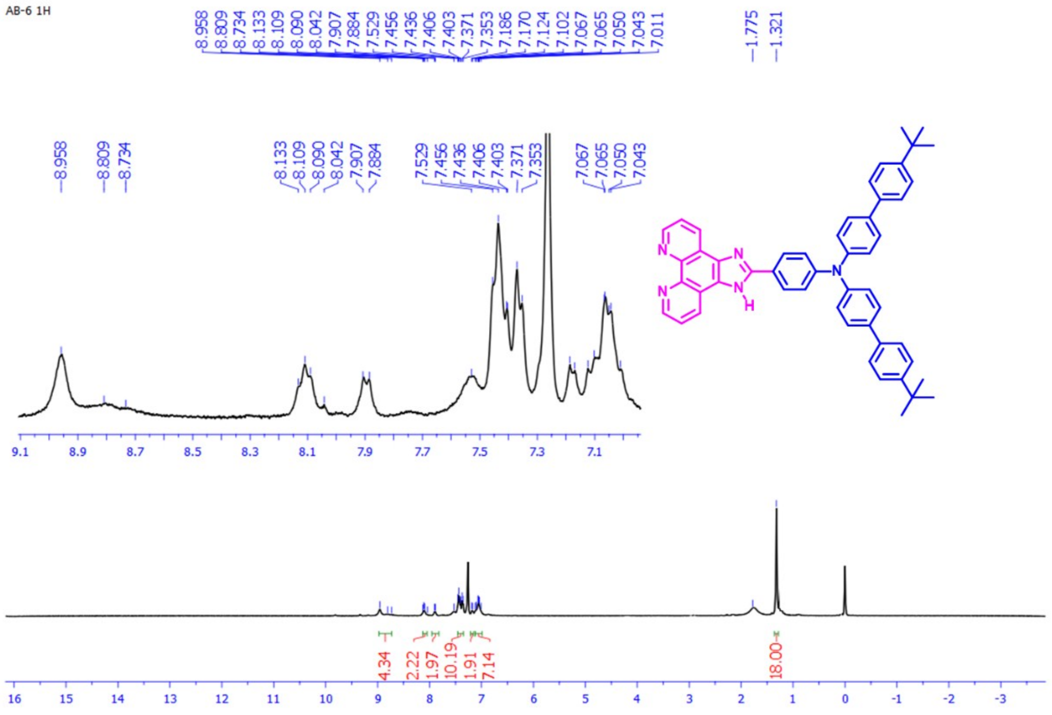

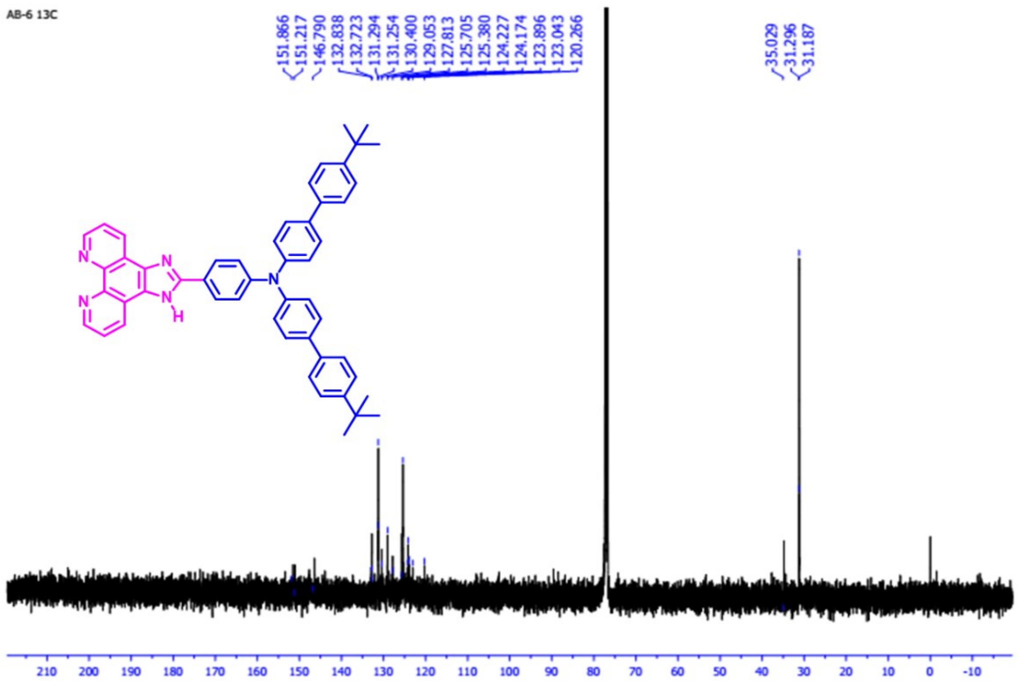

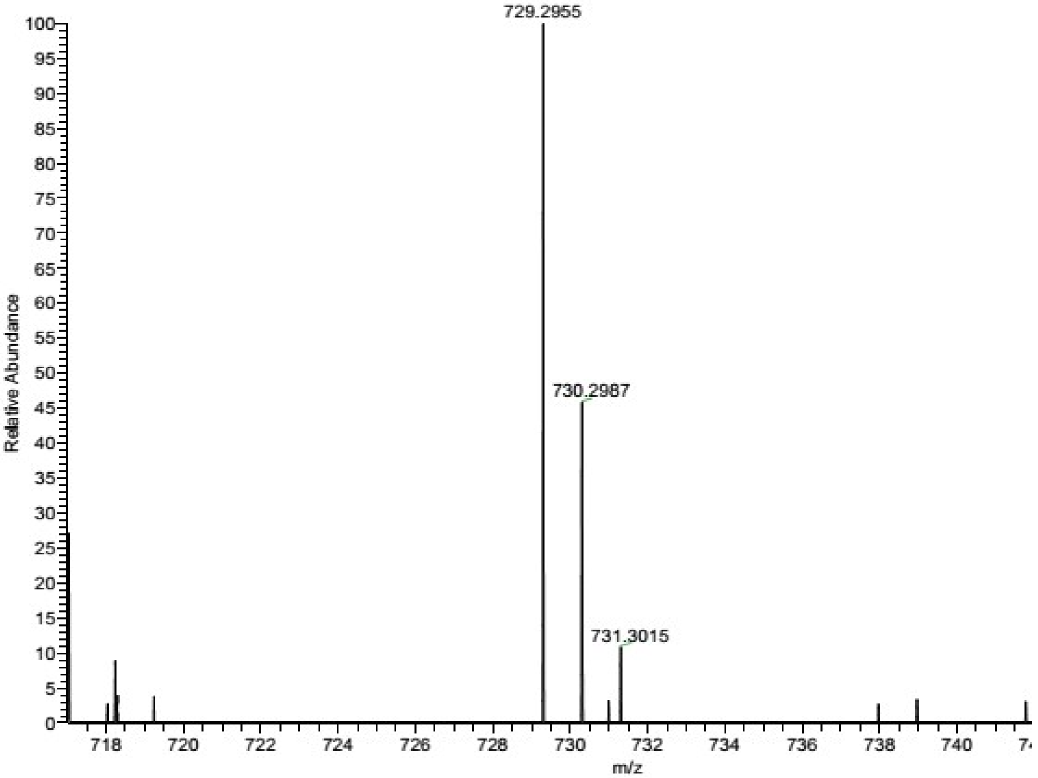

Figure 16. ^{1}H and ^{13}C NMR spectra of compound **6**

Figure S17. HR-mass spectrum of compound **6**

---

[1] (a) G Kresse and J Furthmuller, Phys. Rev. 1996, **54**, 11169 (b) Computat. Mat. Sci. 1996, **6**, 15. (c) G Kresse and D Joubert, Phys. Rev. 1999, B **59**, 1758. (d) [J. Harl and G. Kresse Phys. Rev. Lett. 2009, **103**, 056401. (e) J. Harl, L. Schimka, and G. Kresse Phys. Rev. 2010, **B81**, 115126 (f) M. Dion, H. Rydberg, E. Schröder, D. C. Langreth, and B. I. Lundqvist, Phys. Rev. Lett. 2004, **92**, 246401.
